# Reliable discrimination of type 1 and type 2 diabetes by flow-through leukocyte-endothelium interactions exploiting advanced hydrodynamic detection parameters

**DOI:** 10.1007/s00216-026-06377-6

**Published:** 2026-02-16

**Authors:** Jonathan Hermenejildo, Sandra López-Doménech, María Pelechá-Salvador, Carlos Morillas, Milagros Rocha, Manuel Miró, Enrique Javier Carrasco-Correa

**Affiliations:** 1 Department of Endocrinology and Nutrition, University Hospital Doctor Peset, Foundation for the Promotion of Health and Biomedical Research in the Valencian Region (FISABIO), Valencia, 46017 Spain; 2 CIBEREHD (Centro de Investigación Biomédica en Red de Enfermedades Hepáticas y Digestivas), Madrid, 28029 Spain; 3 FI-TRACE Group, Department of Chemistry, University of the Balearic Islands, Carretera de Valldemossa, km 7.5, Palma, 07122 Spain; 4 CLECEM Group, Department of Analytical Chemistry, Faculty of Chemistry, University of Valencia, Av. Vicent Andrés Estellés, 19, Burjassot, València 46100 Spain

**Keywords:** Diabetes, Leukocyte-endothelial cells interactions, Inflammation, Hydrodynamic variables, Linear discriminant analysis

## Abstract

Type 1 (T1D) and type 2 diabetes (T2D) are both associated with chronic inflammation and endothelial dysfunction, yet their discrimination based on conventional inflammatory biomarkers remains challenging. In this context, a proof-of-concept study exploring whether advanced hydrodynamic descriptors of leukocyte-endothelium interactions under flow conditions might disclose invaluable information to differentiate between diabetic phenotypes is presented. Neutrophil dynamics were investigated using a parallel-plate flow chamber and quantitative video tracking, from which an expanded set of novel hydrodynamic variables encompassing velocity-, acceleration-, rolling-, interaction-, and count-based hydrodynamic parameters was extracted. Supervised machine learning models based on linear discriminant analysis (LDA) were developed and validated using cross-validation strategies. While classical flow parameters and circulating inflammatory biomarkers showed limited discriminatory power, a parsimonious subset of novel hydrodynamic descriptors enabled robust separation between healthy, T1D, and T2D individuals, despite the moderate cohort size (*n* = 10 per group). Weak to moderate correlations with biomarkers indicate that leukocyte dynamics capture complementary functional information beyond static molecular markers. Overall, this approach highlights the potential of flow-based leukocyte dynamics and interpretable machine learning models as a promising functional platform for phenotyping inflammatory microvascular alterations in diabetes.

**Graphical abstract:**

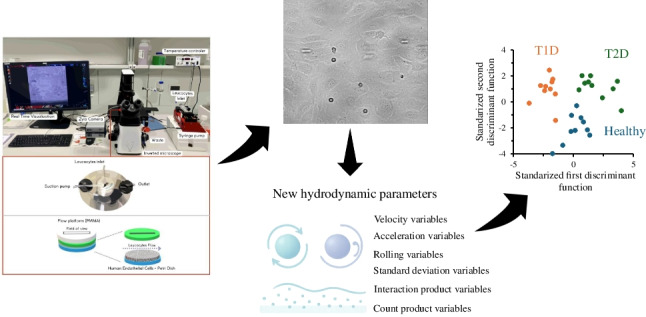

**Supplementary information:**

The online version contains supplementary material available at 10.1007/s00216-026-06377-6.

## Introduction

Diabetes is defined as a chronic condition that affects blood glucose levels. There are two primary types of diabetes mellitus: type 1 diabetes (T1D) and type 2 diabetes (T2D) [1]. Understanding the differences between these two types is crucial for effective management and treatment. T1D accounts for about 5–10% of all diabetes cases. It is an autoimmune condition in which the body’s immune system attacks insulin-producing beta cells in the pancreas [2]. This results in little or no insulin production, requiring patients to rely on external insulin for survival. On the other hand, T2D is much more common, accounting for approximately 90–95% of all diabetes cases. Its representative metabolic disturbances are insulin resistance, hyperglycemia, and altered lipid metabolism [3].

Both diabetes types share underlying mechanisms such as chronic inflammation and vascular damage, leading to complications in both macro- and micro-blood vessels [4]. Inflammation is a key mechanism contributing to atherosclerosis, and leukocytes play a pivotal role in this process. The atherosclerotic process is complex and characterized by narrowing of the arteries that can progress toward occlusion of the vessel or blocking the flow of blood, generating an atherothrombotic event such as myocardial infarction or stroke [5]. Detecting subclinical atherosclerosis is crucial for prognosis and proper treatment. One of the main strategies is identifying the direct relationship between atherosclerotic lesions and conventional cardiovascular risk factors [6]. Coronary artery calcium scoring, semi-automated three-dimensional vascular ultrasound, and intima-media thickness have been more widely explored to predict cardiovascular risk than the direct evaluation of atheroma [7–9]. However, these tests can be invasive for the patient, have high costs, or only explore the coronary territory, overlooking early atherosclerosis.

To improve individual cardiovascular risk predictions, the study of the interaction of leukocytes with the blood vessel wall has been proposed. Leukocytes, such as neutrophils or monocytes, constitute one of the primary defenses of the host against most organisms causing infections, but they also actively contribute to atherogenesis [9, 10]. By releasing reactive oxygen species (ROS) and pro-inflammatory mediators, such as the myeloperoxidase (MPO) enzyme and cytokines such as interleukin (IL)−8 and others, leukocytes promote endothelial dysfunction, amplify local inflammation, and enhance further leukocyte recruitment and migration. These processes contribute to the initiation and progression of atherosclerotic plaques [11] even before the disease becomes clinically apparent and are further exacerbated in individuals with conditions such as diabetes. During this adhesion cascade, leukocytes are initially captured by the endothelium through transient, low-intensity interactions that slow their velocity. This is followed by rolling, firm adhesion, and eventual transmigration into the subendothelial space. These steps are tightly regulated by cellular adhesion molecules (CAMs) such as selectins, integrins, and immunoglobulin superfamily members [12]. Previously, researchers have identified the characteristics and phenotypes of neutrophils that are crucial in immune and inflammatory processes, aiming to assess cardiovascular disease risk and incidence in patients with T1D and T2D [13].

Originally introduced in 1987 by Lawrence et al*.* [14] the parallel-plate flow chamber, simulating physiological hemodynamic conditions, has evolved to be the most widely used tool for investigating leukocyte-endothelium interactions under laminar flow conditions, like those of post-capillary vessels [15, 16]. Over time, improvements in the design enabled the simulation of pulsed flows, the lateral visualization, and the recording of leukocytes using a video camera, all of them proven particularly suitable for the identification of inflammatory events in T1D [17] and for investigating pharmacological interventions in the context of T2D [18, 19]. Various analytical platforms have been tested, such as agarose vessels and glass capillary flow chambers [20–22]. Currently, the parallel flow chambers, as those used in this work, overcome the size limitations of Lawrence’s original chamber [14, 15] as they necessitate fewer cells for the assays due to their smaller size.

The analysis of the video recordings from flow-based tests used to visualize the leukocyte behavior has traditionally been performed manually by many researchers [16–18]. Recent technological advances, including software libraries and other modern tools, have enabled real-time analysis of video frames. They offer reliability and ruggedness for complex analysis and medical diagnostics [23], by minimizing variability and researcher-induced errors during parameter characterization.

This study proposes entirely new hydrodynamic parameters in parallel flow chamber assays for high-throughput differentiation of Healthy vs. T1D and T2D individuals using discriminant analysis based on neutrophil-endothelium interaction data. Early disease detection enables timely preventive measures, potentially averting cardiovascular events. Thus, the integration of advanced analytical tools with biological models offers promise for early cardiovascular risk stratification and the development of more personalized and effective preventive strategies.

## Materials and methods

### Study subjects and sample collection

Patients with T1D (*n* = 10) and T2D (*n* = 10) and Healthy subjects (*n* = 10) were recruited from the Endocrinology and Nutrition Department of the University Hospital Dr. Peset (Valencia, Spain). Patients with diabetes were diagnosed according to the guidelines of the American Diabetes Association. The occurrence of morbid obesity or any other autoimmune, malignant, organic, hematological, inflammatory, or infectious disease represented exclusion criteria. Participants were informed and provided written consent. The protocol was approved by the Clinical Research Ethics Committee from the University Hospital Dr. Peset (ID: 97/23). The study adhered to the ethical principles outlined in the Helsinki Declaration. The data generated and analyzed during the current study, if it is not included in the manuscript, is available from the corresponding author upon request.

Anthropometric parameters of all patients were recorded, and blood was drawn from patients from the median cubital vein in fasting conditions (12 h). The biochemical determinations were performed at the Hospital’s Clinical Analysis Department following standard protocols. Supplementary Table S1 provides a summary of the anthropometric and biochemical characteristics of the study population, including sex distribution, duration of diabetes, and concentrations of glucose, insulin, cholesterol, and triglycerides. The table also reports the percentage of participants receiving each type of treatment within the respective groups.

### Flow cytometric analysis of oxidative and mitochondrial parameters

Markers of ROS production and mitochondrial function were assessed by flow cytometry using an Accuri C6 cytometer (BD Biosciences, Franklin Lakes, NJ, USA). Whole blood (500 µL) was treated with Red Blood Cell Lysis Solution (Miltenyi Biotec, Bergisch Gladbach, Germany; Cat. 130-094-183) to remove erythrocytes and then centrifuged. The resulting cell pellet was resuspended and incubated with 5 µL of APC-conjugated anti-human CD45 antibody (BD Biosciences, San Jose, CA, USA; Cat. 555485) to label leukocytes. Samples were subsequently diluted 1:10 and incubated for 10 min with the appropriate fluorescent probes. Total intracellular ROS (tROS), mitochondrial superoxide (mtROS), and cytosolic superoxide (superoxide) were quantified using 2′,7′-dichlorodihydrofluorescein diacetate (DCFH, 5 µM; Invitrogen, Life Technologies, Eugene, OR, USA; Cat. 11500146), MitoSOX™ Red (MTX, 3 µM; Thermo Fisher Scientific, Waltham, MA, USA; Cat. 11579096), and dihydroethidium (dHE, 5 µM; Invitrogen, Life Technologies, Eugene, OR, USA; Cat. 10530533), respectively. Mitochondrial mass (mtMass) was evaluated using MitoTracker™ Green FM (MTG, 3 µM; Thermo Fisher Scientific, Waltham, MA, USA; Cat. 11589106), while mitochondrial membrane potential (MMP) was assessed using tetramethylrhodamine methyl ester (TMRM, 3 µM; Invitrogen, Life Technologies, Eugene, OR, USA; Cat. I34361). Neutrophils population was identified based on CD45 expression (targeting leukocytes) and forward scatter/side scatter discrimination. For each sample, 10,000 events were recorded.

### Evaluation of MPO, inflammatory mediators, and CAMs in serum

Serum was obtained by collecting blood in gel-separator tubes and centrifuging at 1500 × g for 10 min at 4 °C. Serum levels of myeloperoxidase (MPO), granulocyte macrophage colony–stimulating factor (GMCSF), interferon-gamma (IFN-γ), interleukins (IL-1β, IL-2, IL-4, IL-5, IL-6, IL-7, IL-8, IL-10, IL-12, and IL-13), as well as soluble adhesion molecules, including soluble intercellular adhesion molecule-1 (sICAM-1), soluble vascular cell adhesion molecule-1 (sVCAM-1), soluble P-selectin (sP-selectin), and soluble E-selectin (sE-selectin), were measured using bead-based immunoassay Milliplex MAP kits (Millipore CorpCat. HSTCMAG-28SK, and PPX-05-MX7DTJT, respectively) on a Luminex® 200 analyzer (Thermo Fisher Scientific, Waltham, MA, USA), following the manufacturer’s instructions. 

### Neutrophil isolation

Neutrophils were isolated from anticoagulated whole blood collected in 10 mL EDTA-coated tubes, which are coated with 10.8 mg of EDTA applied as a uniform spray on the interior surface to ensure effective anticoagulation. (Becton, Dickinson and Company, NJ, USA, Cat. 12957666) by means of the MACSxpress® Whole Blood Neutrophil Isolation kit, human (Milteny Biotech, Bergisch Gladbach, Germany, Cat. 130-115-169) following the manufacturer’s instructions.

### Endothelial cell culture

Immortalized human umbilical vein endothelial cells (HUVECs)/TERT2 (ATCC, Manassas, VA, USA, Cat. CRL-4053) were cultured in 25 cm^2^ cell culture flasks. The growth medium for this cell line consisted of Vascular Cell Basal Medium supplemented (92.4% v/v) with Vascular Cell Growth Kit-VEGF (7.5% v/v) and phenol red (0.1% v/v) (all three from ATCC, Manassas, VA, USA, Cat. PCS100030, PCS100041, and PCS999001) and 1% penicillin/streptomycin (Biowest, Nuaillé, France, Cat. MS01TR102C). Confluent endothelial cells (Passaging 3–7) were detached with trypsin-EDTA 1× (Biowest, Nuaillé, France, Cat. MS022G100J) for less than 3 min, washed in Hanks’ balanced salts solution (HBSS) (Biowest, Nuaillé, France, Cat. L0612), and resuspended in 1 mL of fresh medium. Cells were then seeded using a 9 × 10^3^ cells/cm^2^ density in 35-mm Petri dishes (Corning TM, NY, USA, Cat. 430165) and maintained in the incubator at 37 °C in a 5% CO_2_ atmosphere until reaching a confluence of 80–90%. We used the tumor necrosis factor alpha (TNF-α) inflammatory cytokine (1.25 ng/mL, 4 h; Sigma-Aldrich, Millipore Corporation, MA, USA, Cat. SRP3177-50UG) as a positive control for HUVECs.

### Parallel plate flow chamber model

**Fig. 1:**
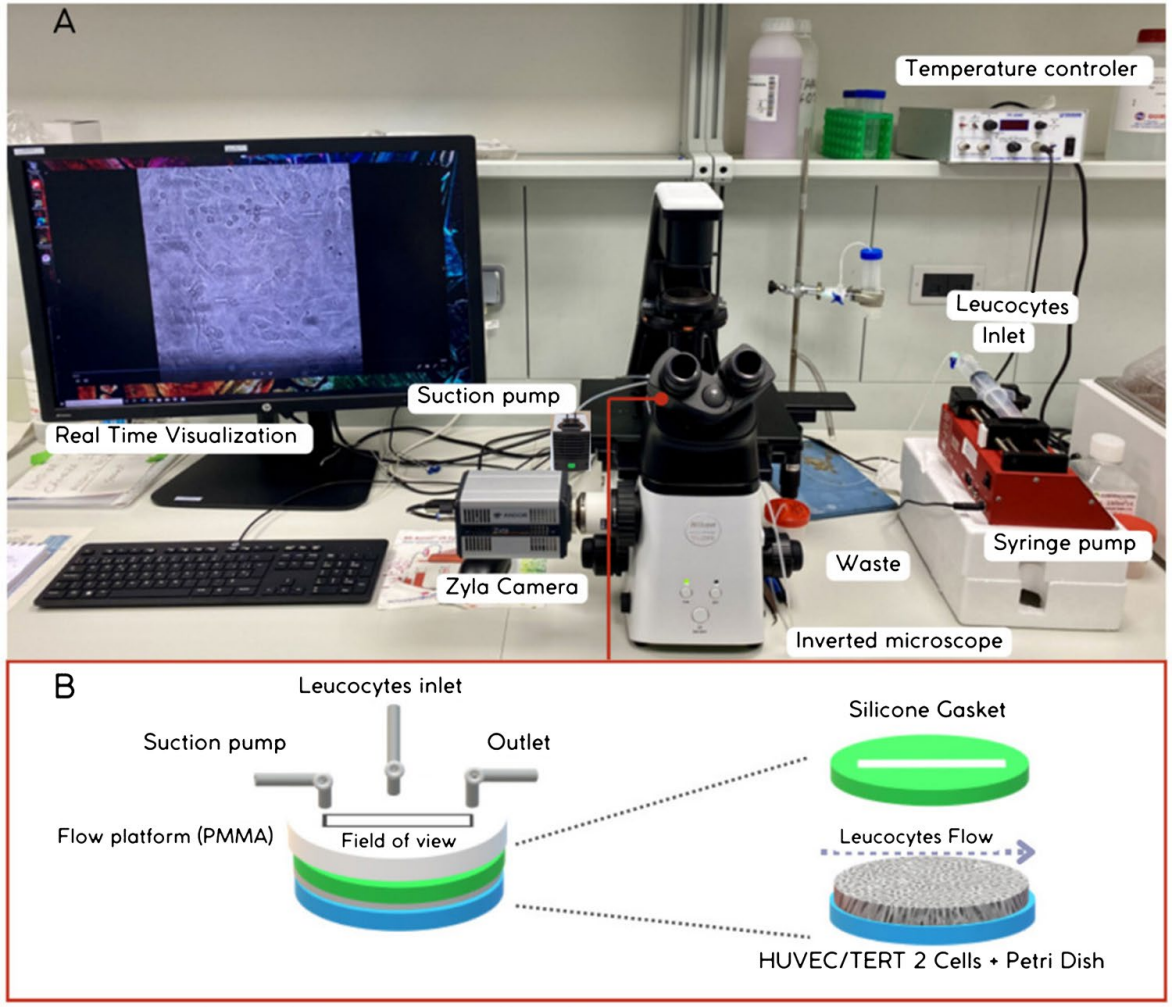
Illustration of the parallel plate flow chamber system (**A**) and flow chamber platform components (**B**)

To evaluate leukocyte-endothelial cell interactions, a parallel plate flow chamber system was herein assembled (Fig. 1). As it can be seen in Fig. 1A, the flow setup is composed of a (i) phase-contrast inverted microscope (Nikon Eclipse TE 2000-S) coupled to a video camera (ZYLA 4.2, Andor, Belfast, UK, Cat. vsc-04170) and to a computer that allows recording and monitoring the events in real time; (ii) controlled perfusion system with temperature regulation (Warner Instruments, MA, USA, Cat. TC-324C); and (iii) vacuum system.

The flow chamber made of poly(methyl methacrylate) (PMMA) contains inlet/outlet ports through which leukocyte cells and liquids are perfused (Fig. 1B). This platform is mounted over the Petri dish covered with the HUVECs by means of a silicone membrane (0.25 mm thick) (GlycoTech System, Gybco, AL, USA, Cat. 31-004) featuring a rectangular window to create the flow chamber of 100 ${\mathrm{mm}}^{2}$. The system assembly is maintained by a vacuum pump (KNF, Freiburg-Munzingen, Germany, Cat. N86KT.18) connected to the vacuum port of the chamber. The inlet port was connected to a polypropylene syringe of 50 mL (Becton, Dickinson and Company, NJ, USA, Cat. 300866) connect to a piston pump (SyringeONE, World Precision Instruments, Hitchin, UK, Cat. NE-100) in a single-pass configuration via a 1.5 mm ID × 3.1 mm OD Silastic tube, generating a perfusion flow rate of 0.3 mL/min. Solutions are preheated before entering the inlet point using a heater controller at 37 °C with a single in-line solution heater (Warner Instruments, MA, USA, Cat. SH-27B). A three-way valve (Discofix C, B. Braun, Melsungen, Germany, Cat. 16494 C) allows switching from one solution to another. All waste generated in the system is collected through the outlet port into a reservoir, whereupon they are pretreated with sodium hypochlorite and kept in the biosafety cabinet for a minimum of 2 h and then disposed of through appropriate drainage systems.

### Leukocyte-endothelium interaction

To mimic the interaction between leukocytes and the endothelial cell layer within blood vessels, thereby simulating physiological flow conditions, an ex vivo model based on the parallel plate flow chamber described above was used. Isolated neutrophils were resuspended (10^6^ cells/mL) in RPMI 1640 media with glutamine supplemented with 10% (v/v) fetal bovine serum (FBS) (Biowest, Nuaillé, France, Cat. FL 34211), 1% (v/v) 100 nM sodium pyruvate (Sigma-Aldrich, Cat. S8636), and 1% (v/v) penicillin/streptomycin (Biowest, Cat. MS01TR102C). The flow chamber was purged with 10 mL of HBSS to remove air bubbles before passing the leukocytes. Neutrophils were then perfused over the HUVEC/TERT2 monolayer for 5 min at a constant flow rate of 0.3 mL/min and maintained at 37 °C using an automatic temperature controller. The entire perfusion of the leukocytes in every sample was recorded in a single field through the monitoring window of the platform using the 40× objective of the microscope and the coupled camera.

Image capture and video recording were performed using NIS-Elements V5.11.03 (Nikon Instruments Inc., Melville, NY, USA), employing the AVI/MP4 acquisition module under standardized settings for all experiments. Videos were acquired in live mode with the following configuration: quality, Max FPS rate, and a fixed duration maximum of 10 min per recording. These parameters ensured reliable frame acquisition and comparable temporal resolution across all samples. Additionally, the camera settings for the Zyla 4.2 sCMOS (Andor) were also maintained identical in every experiment. The camera was operated with a 2 × 2 binning, exposure time of 10 ms, rolling shutter readout mode, 12-bit depth, and gain of 1. These conditions provided a stable signal-to-noise ratio and prevented oversaturation during leukocyte tracking. All videos were saved in uncompressed AVI format and subsequently processed using the analysis pipeline described above.

### Software-based standard tests for characterization of leukocyte-endothelium interactions

Recorded images were analyzed to determine rolling velocity by measuring the time taken by 53 consecutive leukocytes to pass over 100 µm of endothelium. Rolling flux was evaluated by counting leukocytes rolling over a 100 µm^2^ area of HUVEC for more than 1 min, whereas adhesion was assessed by counting leukocytes maintaining firm contact for at least 30 s with the endothelial cells. For cell adhesion quantification, adhered cells were counted in ten randomly selected fields during real-time observation, and the mean value was extrapolated to the total surface area of the Petri dish (8.77 mm^2^). Those determinations were made using the Tracker v6.1.5 software, a video analysis tool based on the Open-Source Physics Java framework [24].

The Tracker software enables calculating and visually displaying the neutrophils kinematics, facilitating detailed motion analysis. The software allows the user to calibrate the video scale to a reference of 327 µm and add a coordinate system with axes *X*, 600 µm and *Y*, 480 µm. This tool also enables tracking leukocytes motion (tracks) in each frame of the video, which is associated with a specific time period. Tracks generated data such as position vs. time and velocity vs. time. The velocity [*v* (*t*) = (*x*(*t* + Δ*t*) − *x*(*t*))/Δ*t*] is calculated using the difference between positions in two consecutive frames, divided by the time interval between those frames.

Although the assay in a parallel-plate flow chamber involves some manual steps that require operator training, multiple methods were established to ensure reproducibility and reduce variability. It is important to note that all procedures related to chamber assembly, perfusion, image capture, and video analysis were performed by the same trained investigator, following the standardized protocol described above. This methodology enabled reliable equipment handling, uniform placement of the silicone gasket, and consistent control over flow, temperature, and bubble removal.

To further enhance reproducibility, the chamber’s geometric parameters (window width and height) were checked before each test, and the perfusion medium and syringes were always maintained at 37 °C. The flow rate was set at 0.3 mL/min using the same calibrated syringe pump, and recordings were made with standard microscope settings. Video analysis in the Tracker software was performed following an identical set of steps for all samples, including spatial calibration, criteria for particle tracking, and standardized operational definitions for filming and adhesion.

### Statistical and multivariate analysis

Sample size estimation was performed using the GRANMO calculator (version 8.0) to ensure a statistical power of 80% to detect significant (*p* < 0.05, two-sided) differences of at least 300 units in the primary outcome (rolling velocity), assuming a common standard deviation of 200 units across the three study groups. Under these assumptions, a minimum of 10 subjects per group was required. A loss-to-follow-up rate of 0% was assumed.

Statistical analyses based on the variables examined throughout the study were generated using the IBM SPSS statistics software v.22 (IBM Corp., Armonk, NY, USA). Group comparisons were performed using one-way analysis of variance (ANOVA) followed by Tukey’s honestly significant difference (HSD) post hoc test. Statistical significance was set at *p* < 0.05. Principal component analysis (PCA) was applied to standardized hydrodynamic variables using the correlation matrix and principal component extraction. Components with eigenvalues > 1 were retained for exploratory purposes. Varimax rotation was applied to facilitate interpretation. PCA was used exclusively as an unsupervised exploratory tool. Linear discriminant analysis (LDA) was employed as the supervised multivariate classification method. Variable selection was performed using a stepwise procedure (*F*_in_ = 3.84; *F*_out_ = 2.71). The validation of the models was done by the leave-one-out (LOO) methodology, which consists of iteratively removing one observation from the dataset, training the model on the remaining data, and then testing it on the excluded observation. This process is repeated for each data point, providing an unbiased estimate of the model’s predictive performance, which is particularly valuable given the small sample size in the present study. In order to study the different models developed throughout the manuscript, a key statistic used in multivariate analysis, Wilks’ lambda (*λ*_*w*_), will be used, which measures the ratio of within-group variability to total variability, with values ranging from 0 to 1. A lower lambda indicates that most of the variance in the data is explained by differences between groups rather than within them, suggesting that the model has good discriminatory power.

## Results and discussion

### Standard parameters for evaluation of leukocyte-endothelium interactions

Leukocyte-endothelium interactions are classically evaluated by measuring specific parameters using the video software Tracker v6.1.5, as described in section “Parallel plate flow chamber model.” In this context, three parameters are typically assessed: rolling velocity, rolling flux, and adhesion. These are widely used to investigate neutrophil interactions with blood vessels, as low rolling velocities, high rolling flux, and a large number of adhered cells reflect stronger interaction with the endothelium, representing the initial steps of leukocyte capture and migration to sites of inflammation. In the context of chronic inflammatory diseases, previous works evaluating dynamic leukocyte-endothelium interactions merely compared either T1D or T2D patients versus control subjects via *t*-tests or ANOVA and HSD Tukey post hoc tests using large groups (*N* > 50) [16–18], yet the discrimination of T1D vs. T2D has not been feasible as of yet.

**Fig. 2:**
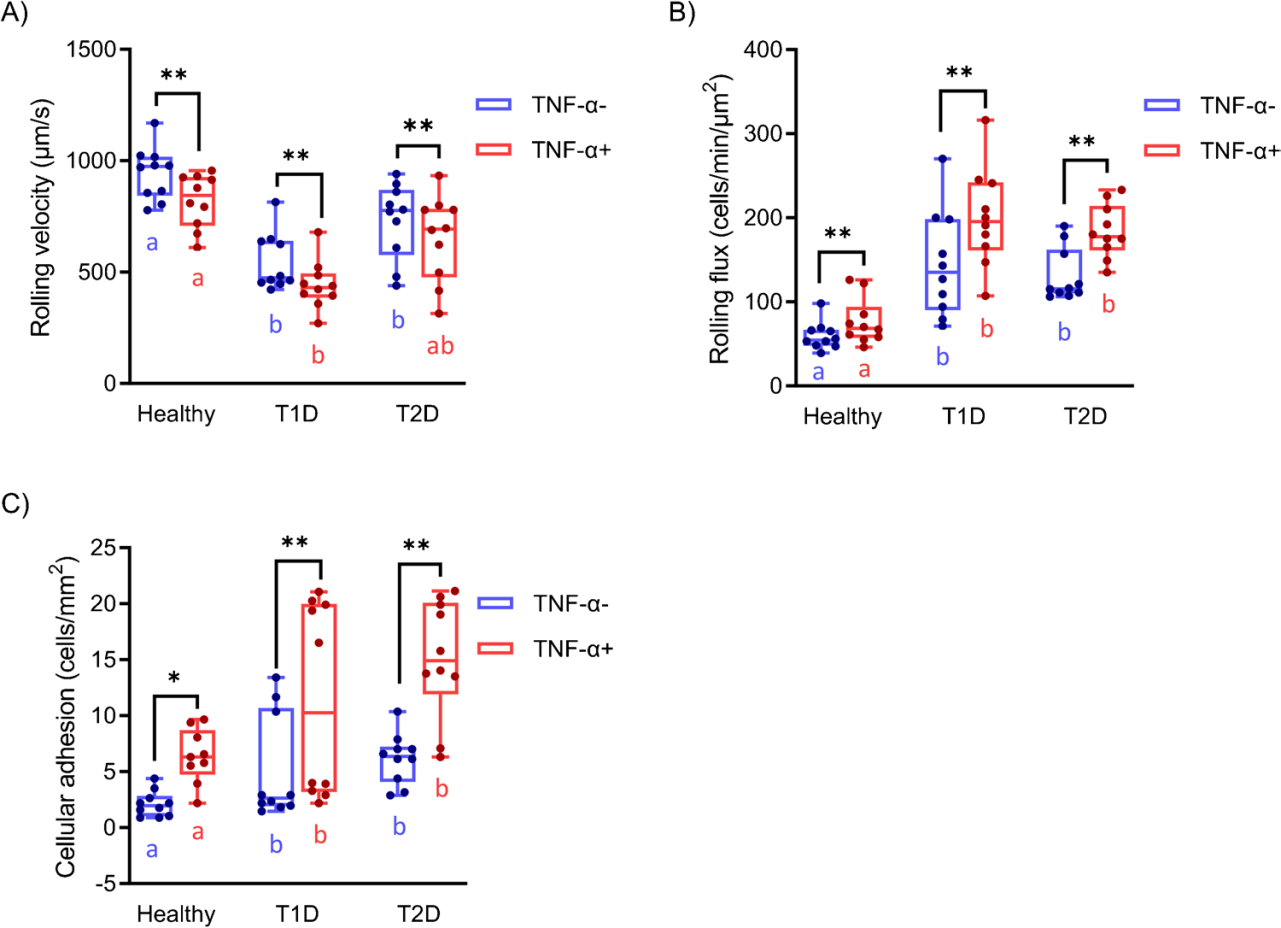
Comparison of classic leukocyte-endothelium interaction parameters across Healthy, T1D, and T2D groups. Data are presented as box and whisker plots (min–max), with all individual data points shown. Blue boxplots represent leukocyte interactions on untreated HUVECs (without TNF-α), whereas red boxplots represent interactions on TNF-α-treated HUVECs (with TNF-α). Statistical comparisons were performed using one‐way ANOVA followed by the HSD Tukey post hoc test, as appropriate. *p < 0.05; **p < 0.01. Different superscript letters indicate significant differences among groups. Groups sharing the same superscript letter are not significantly different from each other (*p* > 0.05), whereas groups with different letters show statistically significant differences (*p* < 0.05)

Anthropometric and biochemical characteristics and current treatments of the study population are detailed in Table S1. Importantly, T1D and T2D participants exhibited comparable diabetes duration, a relevant consideration given that cumulative exposure to hyperglycemia is a key determinant of cardiovascular risk. Group differences among Healthy, T1D, and T2D participants were assessed with adjustment for age as a covariate. Table S2 presents (i) rolling velocity, (ii) rolling flux, and (iii) adhesion values measured in all study participants under two conditions: with and without TNF-α stimulation of HUVECs. Analysis of group differences (Fig. 2) showed that, under basal conditions (absence of TNF-α), the T1D group exhibited reduced rolling velocity (Fig. 2A), whereas both the T1D and T2D groups showed increased rolling flux (Fig. 2B), with no significant differences in leukocyte adhesion compared with Healthy subjects (Fig. 2C). Following TNF-α stimulation of endothelial cells, rolling velocity was further reduced in T1D patients (Fig. 2A), while rolling flux increased in both T1D and T2D groups compared with Healthy subjects (Fig. 2B). In contrast, leukocyte adhesion increased exclusively in T2D patients (Fig. 2C). Additionally, TNF-α significantly reduced rolling velocity in T1D and increased leukocyte adhesion in T2D patients.

The potential statistical differences between several groups were evaluated through ANOVA analyses for the various classical parameters. A post hoc test, the HSD Tukey test, was applied to determine which groups differ from one another. As shown in Tables S3, S4, and S5, statistically significant differences were observed in just a few groups. For rolling velocity (Table S3), the ANOVA *p*-value obtained was 6·10^–10^, indicating statistically significant differences among the groups. Post hoc analysis revealed significant differences between the T1D and T2D groups, whereas no statistically significant differences were observed between Healthy subjects and T2D patients. Consequently, using the rolling velocity as a parameter would result in an unacceptable rate of misclassification in a clinical context. Similar trends were observed for the TNF-α-activated experiments, confirming that classical univariate parameters, despite showing statistical significance in specific pairwise comparisons, are insufficient for robust discrimination among all groups. Regarding the rolling flux parameter, an ANOVA *p*-value of 1.57·10^–7^ (< 0.05) indicates significant differences among the groups. However, the Tukey’s HSD post hoc test (Table S4) revealed significant differences only between Healthy and Healthy+TNF-α against the others and T1D+TNF-α vs. T2D. This suggests that the rolling parameter may not be useful for effective discrimination between T1D and T2D. Even fewer differences were observed for the adhesion parameter (Table S5). Although ANOVA indicated significant differences among groups (*p* = 1.17·10^–6^), none of the important pairs showed statistically significant differences in the results throughout the post hoc analysis.

Given the limited discriminatory power of the individual standard parameters, machine learning by LDA is proposed herein as alternative to attempt classification between groups. LDA enables the combination of multiple parameters into a single predictive model, which might capture complex patterns and interactions that may not be visualized when analyzing parameters individually, thereby improving classification accuracy and ruggedness. In this regard, LDA models were developed for different group comparisons, as shown in Table S6. Binary (1 vs. 1), ternary (1 vs. 1 vs. 1), and full multi-class models were constructed using classical parameters: rolling velocity, rolling flux and adhesion. Although some binary models achieved acceptable classification performance, particularly those comparing Healthy vs. Healthy+TNF-α (*λ*_*w*_ = 0.304; 95% classification accuracy and the same value for the cross-validation accuracy), the majority of the LDA models failed to effectively discriminate between T1D and T2D groups. For example, the T1D vs. T2D model yielded a high *λ*_*w*_ of 0.582 (indicating weak discrimination), and cross-validation accuracy was only 75% for both groups. Similarly, when attempting to distinguish diabetic patients from healthy individuals, the performance was poor despite the low *λ*_*w*_ values that suggested potential separation. In the three-class model (Healthy vs. T1D vs. T2D), the *λ*_*w*_ value was as high as 0.803, with classification accuracies of about 80%. In the most complex model involving all six groups, *λ*_*w*_ increased further to 0.927, and only the Healthy group maintained high cross-classification accuracy (90%), while the remaining diabetic and TNF-α groups, especially T2D and T1D+TNF-α, exhibited very poor cross-validation results (as low as 50% and 30%, respectively). These results suggest that, with the current dataset and sample size, it may not be possible to clearly differentiate between these groups. This limitation highlights the need for additional parameters to improve discriminatory power.

Beyond the pro-adherent phenotype of circulating leukocytes described above, the endothelial dysfunction represents the complementary arm of the leukocyte-endothelium interaction axis. In diabetes, endothelial activation is associated with increased expression and shedding of CAMs, thereby promoting leukocyte capture, rolling, and transmigration. To comprehensively characterize the endothelial inflammatory component, intracellular ROS production and mitochondrial function markers in neutrophils, together with circulating biomarkers, were analyzed (Table S7), including tROS, mtROS, superoxide anion, mtMass, MMP, MPO (a neutrophil-derived pro-oxidant enzyme), CAMs involved in endothelial activation and leukocyte recruitment (sP-selectin, sE-selectin, sICAM-1, and sVCAM-1), and cytokine-mediated inflammatory signaling, encompassing pro-inflammatory and chemotactic mediators (IL-1β, IL-2, IL-6, IL-8, IL-12, IFN-γ, and GM-CSF), regulatory cytokines (IL-10), and Th2-associated mediators (IL-4, IL-5, IL-7, IL-13). As a complementary analysis and following the same approach used for the classical variables, several group comparisons for circulating biomarkers were also performed using one-way ANOVA followed by Tukey’s HSD post hoc test in order to obtain a more global overview of differences among the study groups, as depicted in Fig. S1. As summarized in Table S8, MPO (Fig. S1F, ANOVA *p* = 0.048), IFN-γ (Fig. S1L, ANOVA *p* = 0.007), IL-1β (Fig. S1N, ANOVA *p* = 0.037), and IL-2 (Fig. S1T, ANOVA *p* = 0.044) showed statistically significant differences between groups. Specifically, MPO exhibited significant differences between the Healthy and T1D groups (*p* = 0.044). In contrast, IFN-γ (*p* = 0.005), IL-1β (*p* = 0.028), and IL-2 (*p* = 0.040) showed significant differences between T1D and T2D, while none of these markers differed significantly between either diabetic group and the Healthy group. Overall, these results indicate that, when considered individually, these biomarkers lack sufficient discriminatory power to reliably differentiate all study groups. Specifically, MPO fails to distinguish between T1D and T2D, whereas IFN-γ, IL-1β, and IL-2 differentiate between diabetic subtypes but do not clearly separate diabetic patients from healthy individuals, highlighting limitations in sensitivity and specificity when used as single markers.

To further explore the relationship between biochemical markers and the microvascular phenotype, Pearson and Spearman correlation analyses were performed between all biochemical parameters and the three flow-related classical variables (rolling velocity, rolling flux, and adhesion). However, Pearson coefficients with good correlation (> 0.3) and *p*-values lower than 0.05, apart between classical hydrodynamic variables (*r* between −0.503 and −0.6, with *p*-value < 0.05), were found only for rolling velocity vs. IFN-γ (*r* = −0.387, *p* < 0.05) and rolling flux vs. IL-12 (*r* = −0.4, *p* < 0.05). The other circulating variables showed correlations lower than 0.25. Therefore, Spearman resulting correlation coefficients were also calculated and are summarized in Fig. S2. Overall, the data analysis revealed distinct correlation patterns depending on the flow-related parameter considered. Rolling velocity showed predominantly negative associations with several inflammatory and endothelial activation markers, most notably MPO (*ρ* =  − 0.448, *p* < 0.05), sE-selectin (*ρ* =  − 0.371, *p* < 0.05), and IL-8 (*ρ* =  − 0.326, *p* < 0.05), suggesting that increased inflammatory activity is associated with reduced leukocyte rolling velocity. In contrast, rolling flux exhibited positive correlations with selected inflammatory markers, including MPO (*ρ* = 0.416, *p* < 0.05) and IL-8 (*ρ* = 0.429, *p* < 0.05), as well as a strong positive association with adhesion (*ρ* = 0.679, *p* < 0.001), indicating that higher leukocyte recruitment is linked to increased inflammatory burden. Adhesion itself was negatively correlated with mtMass (*ρ *=  − 0.465, *p* < 0.05), pointing to a potential relationship between cellular metabolic status and firm leukocyte attachment. Importantly, although several statistically significant associations were identified, no single biochemical marker showed a consistent and strong correlation across all flow-related parameters. This finding indicates that individual biomarkers capture only partial aspects of the microvascular dysfunction and are insufficient to fully explain the observed flow behavior independently. Taken together, these results suggest that the inflammatory and endothelial activation processes reflected by biochemical markers are functionally linked to leukocyte-endothelium interactions, but in a multifactorial manner. This observation provides a mechanistic rationale for subsequently evaluating multivariate classification approaches. Herein, LDA-based machine learning will be assessed using biochemical markers alone and in combination with classical flow-related parameters.

LDA revealed that circulating biomarkers alone are not sufficient to reliably classify Healthy, T1D, and T2D groups. Even with a multivariate model including seven biomarkers (MPO, superoxide, sE-selectin, sP-selectin, tROS, mtMass, and IFN-γ), the leave-one-out cross-validation resulted in only 53.3% of correctly classified cases. These results demonstrate that, when considered independently from functional parameters, circulating biomarkers lack adequate predictive power to accurately assign subjects to their corresponding clinical group. Consequently, the integration of biochemical markers with hydrodynamic classical parameters was explored in order to evaluate whether combined models could improve classification performance. For this purpose, the previously established model for classifying Healthy, T1D, and T2D groups based on hydrodynamic classical variables was further evaluated by individually incorporating circulating biomarkers that showed statistically significant group differences (MPO, sICAM-1, sVCAM-1, sE-selectin, IL-8, IFN-γ, IL-1β, and IL-2). In the new models, *λ*_*w*_ decreased to values in the range of 0.680–0.783 compared to 0.803 for the model based solely on flow-related parameters. Although the inclusion of these biomarkers is expected to enhance the discriminatory power of the model, the opposite effect was in fact observed. In several cases, the leave-one-out cross-validation accuracy dropped to values as low as 50%. The decrease in Wilks’ lambda was thus not accompanied by improved cross-validation performance, indicating overfitting and limited added value of individual biomarkers. Concretely, this behavior may be explained by the partial correlation observed between these biochemical markers and classical hydrodynamic parameters, indicating overlapping information and limited additional discriminatory value. Consequently, none of the extended models outperformed the original hydrodynamic model, highlighting the ruggedness and superior discriminatory capability of classical flow-related parameters for group classification. Overall, our findings confirm that leukocyte-endothelial cell interactions and endothelial dysfunction are altered in patients with diabetes. However, classical parameters alone are not sufficient to fully discriminate Healthy, T1D, and T2D in our dataset with the current sample size. Additionally, the LDA results highlight that despite the low *λ*_*w*_ values obtained in some models (suggesting some degree of separation in the feature space), the classical parameters are not sufficiently informative to enable accurate classification, particularly between T1D and T2D, or between diabetic and non-diabetic individuals. This limitation underscores the need for additional, more sensitive hydrodynamic parameters to improve the discriminatory power between diabetic phenotypes.

### Selection of novel leukocyte hydrodynamic variables extracted from video recordings

Currently, the analysis of leukocyte behavior through video recordings focuses on just a few parameters, such as rolling velocity, rolling flux, and adhesion, as indicated above. Additionally, it has been demonstrated in the previous section that the use of circulating biomarkers, alone or in combination with classical hydrodynamic parameters, does not suffice for a reliable classification system. However, the advent of specialized software enables obtaining entirely new hydrodynamic parameters that might provide additional insights into the dynamic and kinetic behavior of leukocytes. This technological advancement enables a more comprehensive and quantitative analysis, facilitating a deeper understanding of leukocyte interactions. In fact, by analyzing leukocyte dynamics, which are closely associated with inflammatory events, it is possible to obtain relevant information on the underlying pathological processes, facilitating early detection of cardiovascular disease, risk stratification, and development of tailored preventive and therapeutic strategies for diabetes and subclinical atherosclerosis. Hence, video recordings of leukocyte behavior might provide invaluable data for classifying patients with varied health conditions, particularly those related to inflammatory and cardiovascular diseases based on the underlying leukocyte-endothelium interactions. In this context, novel experimental variables are amenable to be retrieved from video recordings and can be divided into six groups: (i) velocity variables; (ii) acceleration variables; (iii) rolling variables; (iv) standard deviation variables; (v) interaction product variables; and (vi) count variables. Table 1 shows a summary of all new variables used throughout this work and definitions thereof.

**Table 1:** Description of the novel hydrodynamic variables explored in this work for developing reliable classification methods

Variable	Description
Velocity variables	
${v}_{x}^{\mathrm{ave}}$	Absolute average velocity of all leukocytes along the *X*-axis
${v}_{x}^{\mathrm{med}}$	Median velocity of leukocytes along the *X*-axis
${v}_{x}^{{\#}_{1}-{\#}_{2}}$	Average velocities of leukocytes in specific ranges (#_1_–#_2_)
Acceleration variables	
${a}_{x}^{\mathrm{ave}}$	Absolute average acceleration of all leukocytes along the *X*-axis
${a}_{x}^{\mathrm{med}}$	Median acceleration of leukocytes along the *X*-axis
${v}_{x}^{\mathrm{ave}}/{a}_{x}^{\mathrm{ave}}$	Ratio of average velocity to average acceleration
${v}_{x}^{\mathrm{med}}/{a}_{x}^{\mathrm{med}}$	Ratio of median velocity to median acceleration
${v}_{x}^{\mathrm{ave}}\times {a}_{x}^{\mathrm{ave}}$	Product of average velocity and average acceleration
${v}_{x}^{\mathrm{med}}\times {a}_{x}^{\mathrm{med}}$	Product of median velocity and median acceleration
${a}_{x}^{{\#}_{1}-{\#}_{2}}$	Average accelerations of leukocytes in specific ranges (#_1_–#_2_)
Rolling variables	
${R}_{\mathrm{ave}}^{+}$	Average number of positive rollings (accelerations) per leukocyte
${R}_{\mathrm{ave}}^{-}$	Average number of negative rollings (decelerations) per leukocyte
${R}_{\mathrm{ave}}^{+}/{R}_{\mathrm{ave}}^{-}$	Ratio of positive to negative rollings
Standard deviation variables	
${s}_{{v}_{x}}$	Standard deviation of leukocyte velocity along the *X*-axis
${s}_{{a}_{x}}$	Standard deviation of leukocyte acceleration along the *X*-axis
${s}_{{v}_{x}}/{s}_{{a}_{x}}$	Ratio of velocity standard deviation to acceleration standard deviation
${s}_{{v}_{x}}\times {s}_{{a}_{x}}$	Product of velocity and acceleration standard deviations
${v}_{x}^{\mathrm{ave}}/{s}_{{v}_{x}}$	Ratio of average velocity to its standard deviation
${a}_{x}^{\mathrm{ave}}/{s}_{{a}_{x}}$	Ratio of average acceleration to its standard deviation
Interaction product variables	
${v}_{x}^{{\#}_{1}-{\#}_{2}}\times {v}_{x}^{{\#}_{1}-{\#}_{2}}$	Product of two velocity ranges
${a}_{x}^{{\#}_{1}-{\#}_{2}}\times {a}_{x}^{{\#}_{1}-{\#}_{2}}$	Product of two acceleration ranges
Count variables	
${N}_{{v}_{x}}^{{\#}_{1}-{\#}_{2}}$	Number of leukocytes in each velocity range
${N}_{{a}_{x}}^{{\#}_{1}-{\#}_{2}}$	Number of leukocytes in each acceleration range

Within the velocity variables, velocities of leukocytes along the *X*-axis of the video recordings might provide insight into how quickly leukocytes are moving, thus indicating the level of inflammatory response or leukocyte activation across different patient groups. In this group, the absolute mean velocity of all leukocytes (${v}_{x}^{\mathrm{ave}}$), the median velocity (${v}_{x}^{\mathrm{med}}$), and the average velocities at specific ranges (${v}_{x}^{{\#}_{1}-{\#}_{2}}$, where #_1_–#_2_ indicates the range of velocities) can be assessed. In the first case, ${v}_{x}^{\mathrm{ave}}$ gives an overall picture of how active the immune response is in a given patient. Low overall velocities might indicate heightened immune activity, which can be associated with inflammation. The variable ${v}_{x}^{\mathrm{med}}$ of leukocytes along the *X*-axis offers a reliable measure of the central tendency within their movement speeds, which can be crucial for understanding variations in leukocyte activity. Regarding ${v}_{x}^{{\#}_{1}-{\#}_{2}}$, this variable breaks down the velocities into specific ranges to gain further data on mobility differences against leukocytes. In this sense, patients with different inflammations might show different patterns in these ranges. In the specific study carried out in this work related to the classification of diabetes-affected or non-affected patients, the velocities were ranged into 4 groups for #_1_–#_2_ in the ranges 0–500, 500–1000, 1000–1500, and 1500-$\infty$ μm s^–1^.

Acceleration metrics offer additional insights into the dynamic behavior of leukocytes. The average acceleration (${a}_{x}^{\mathrm{ave}}$), the median acceleration (${a}_{x}^{\mathrm{med}}$), and the parameters involving velocity and acceleration, ratios (${v}_{x}^{\mathrm{ave}}/{a}_{x}^{\mathrm{ave}}$ and ${v}_{x}^{\mathrm{med}}/{a}_{x}^{\mathrm{med}}$) or products (${v}_{x}^{\mathrm{ave}}\times {a}_{x}^{\mathrm{ave}}$ and ${v}_{x}^{\mathrm{med}}\times {a}_{x}^{\mathrm{med}}$), might help understanding how quickly leukocytes are changing their speed. There are currently few studies specifically investigating leukocyte acceleration, because velocity has traditionally been one of the primary hydrodynamic parameters used to characterize leukocyte-endothelium interactions [16–18]. Nevertheless, Hafezi-Moghadam et al*.* reported the occurrence of transient accelerations and decelerations, described as “jerkins” acceleration, that may have relevant biological implications in the process of leukocyte recruitment and cell migration [25, 26]. Changes in acceleration/deceleration may reflect dynamic shifts in the chemical affinity between adhesion molecules such as integrins, selectins, and immunoglobulins of the leukocyte and their ligands on endothelium [12, 26]. Thus, variations in rolling velocity throughout time could indicate transitions between low- and high-affinity interactions, which ultimately might lead to change from rolling to firm adhesion. The ${a}_{x}^{\mathrm{ave}}$ indicates how quickly leukocytes are speeding up or slowing down and might give insight into the actual inflammation level. In the case of ${a}_{x}^{\mathrm{med}}$, a central measure of how leukocytes' speeds are changing is provided. This might help identifying typical acceleration patterns within a patient’s leukocyte population. The ratio ${v}_{x}^{\mathrm{ave}}/{a}_{x}^{\mathrm{ave}}$ provides insight into how velocity and acceleration correlate. Ratios significantly lower or higher than 1 might suggest a strong and swift leukocyte response, possibly pointing to inflammation. The product ${v}_{x}^{\mathrm{ave}}\times {a}_{x}^{\mathrm{ave}}$ indicates the overall momentum of leukocytes, with lower values potentially correlating with aggressive inflammatory responses. ${v}_{x}^{\mathrm{med}}/{a}_{x}^{\mathrm{med}}$ and ${v}_{x}^{\mathrm{med}}\times {a}_{x}^{\mathrm{med}}$ help comparing the central tendency of velocity to that of acceleration. It is useful for highlighting whether changes in velocity are consistent with acceleration patterns, giving clues about the dynamics of leukocyte interactions with the endothelium. Additionally, akin to velocity, average acceleration is calculated at specific ranges (${a}_{x}^{{\#}_{1}-{\#}_{2}}$, where #_1_–#_2_ indicates the range of accelerations). In this case, the accelerations were ranged into four groups for #_1_–#_2_ in the ranges (–∞)–(–5000), (–5000)–0, 0–5000, and 5000-$\infty$ μm^2^ s^–1^. Patients with different diabetes-affected problems might show different patterns in these ranges. For example, a higher proportion of leukocytes with moderate accelerations might be seen in T1D diabetes, indicating a stable but persistent response.

Another important group of variables are the rolling flux variables, which measure the average number of positive and negative rolling events that correspond to leukocyte acceleration or deceleration during their interactions with the vessel wall. The number of rolling events are measured photogram-by-photogram for each leukocyte, looking for accelerations and decelerations. Three interesting variables can be obtained from these data: ${R}_{\mathrm{ave}}^{+}$, ${R}_{\mathrm{ave}}^{-}$, and ${R}_{\mathrm{ave}}^{+}/{R}_{\mathrm{ave}}^{-}$, which are the average number of positive rolling events (number of accelerations) per leukocyte, the average number of negative rolling events (number of decelerations) per leukocyte and the ratio of the positive to negative rolling events, respectively. In this case, high values of ${R}_{\mathrm{ave}}^{+}$ or ${R}_{\mathrm{ave}}^{-}$ might suggest active detachment or adhesion of leukocytes to the vessel wall, respectively. Hence, these parameters could be related to different inflammatory levels or diseases, such as T1D and T2D. Regarding the ratio ${R}_{\mathrm{ave}}^{+}/{R}_{\mathrm{ave}}^{-}$, which shows the balance between leukocyte detachment and adhesion, a high ratio might indicate active inflammation with ongoing leukocyte detachment, while a low ratio could suggest persistence of high levels of inflammation.

The heterogeneity of leukocyte dynamics might reveal important information about the behavior of leukocytes flowing over the endothelium. In this sense, higher variability might indicate more erratic leukocyte activity, which could be linked to more severe or unstable disease conditions. Standard deviation metrics from velocity (${s}_{{v}_{x}}$) and acceleration (${s}_{{a}_{x}}$) quantify the variability in velocity and acceleration of the leukocyte population. Additionally, the relation between these two variables (${s}_{{v}_{x}}/{s}_{{a}_{x}}$ and ${s}_{{v}_{x}}\times {s}_{{a}_{x}}$) and the relationship with the average values of velocity (${v}_{x}^{\mathrm{ave}}/{s}_{{v}_{x}}$) and acceleration (${a}_{x}^{\mathrm{ave}}/{s}_{{a}_{x}}$) have been also examined as potential useful parameters. The metrics ${s}_{{v}_{x}}$ and ${s}_{{a}_{x}}$ of leukocyte population show how variable the leukocyte speeds and accelerations are. In the case of the velocity and acceleration, high standard deviation might indicate a heterogeneous inflammatory response, due to variability related with the interactions of leukocyte with the endothelium, which can be linked to unstable or mixed pathologies. The ${s}_{{v}_{x}}/{s}_{{a}_{x}}$ and ${s}_{{v}_{x}}\times {s}_{{a}_{x}}$ might provide insight into the relative consistency of leukocyte movement patterns. The relationships between velocity/acceleration and their respective standard deviations indicate whether velocity and acceleration patterns are consistent or erratic or caused by a few outlier leukocytes moving very low or very fast.

The interaction product variables represent the product of two velocities or two accelerations in different ranges, reflecting the interaction between leukocytes moving at different speeds/accelerations. They might help identifying complex patterns of leukocyte behavior that are indicative of specific pathological conditions. In this work, six product interaction variables for velocity and six for acceleration that compile all the possibility products of the four ${v}_{x}^{{\#}_{1}-{\#}_{2}}$ ranges and the four ${a}_{x}^{{\#}_{1}-{\#}_{2}}$ ranges, respectively, were selected for further studies. These variables were ${v}_{x}^{0-500}\times {v}_{x}^{500-1000}$; ${v}_{x}^{0-500}\times {v}_{x}^{1000-1500}$; ${v}_{x}^{0-500}\times {v}_{x}^{1500-\infty }$; ${v}_{x}^{500-1000}\times {v}_{x}^{1000-1500}$; ${v}_{x}^{500-1000}\times {v}_{x}^{1500-\infty }$; ${v}_{x}^{1000-1500}\times {v}_{x}^{1500-\infty }$; ${a}_{x}^{(-\infty )-(-5000)}\times {a}_{x}^{(-5000)-0}$; ${a}_{x}^{(-\infty )-(-5000)}\times {a}_{x}^{0-5000}$; ${a}_{x}^{(-\infty )-(-5000)}\times {a}_{x}^{5000-\infty }$; ${a}_{x}^{(-5000)-0}\times {a}_{x}^{0-5000}$; ${a}_{x}^{(-5000)-0}\times {a}_{x}^{5000-\infty }$; and ${a}_{x}^{0-5000}\times {a}_{x}^{5000-\infty }$. For instance, ${v}_{x}^{0-500}\times {v}_{x}^{500-1000}$ might show how slower-moving leukocytes interact with moderately fast ones. Such interactions can be critical for understanding mixed responses in complex inflammatory environments.

Count variables have been also taken into account in this work. These counts indicate the number of leukocytes falling within specific velocity and acceleration ranges and might serve for signalling the level of interaction between the leukocytes and the endothelium. Differences across these counts might help distinguishing between conditions of low against high leukocyte activity. In this context, ${N}_{{v}_{x}}^{{\#}_{1}-{\#}_{2}}$ and ${N}_{{a}_{x}}^{{\#}_{1}-{\#}_{2}}$ have been split into four groups each using the same ranges than those of velocities (0–500, 500–1000, 1000–1500, and 1500–∞ μm s^–1^) and accelerations ((–∞)–(–5000), (–5000)–0, 0–5000, and 5000–∞ μm^2^ s^–1^). Both cases might help quantifying the distribution of leukocyte speeds and acceleration in a given patient, which might be related to particular inflammatory profile or pathological condition.

Other variables can be obtained from the video recordings, such as those related to directional movements in axes other than *X*, such as *Y* and *Z*. These examinations will result in a number of variables similar to all those already mentioned but add new ones, such as the angular velocity (rate of change of the direction of leukocyte movement) and the angular acceleration and deceleration (rate of change of angular velocity). Nevertheless, the leukocyte tends to travel in a laminar flow regime in the fluidic setup; therefore, negligible movement in axes other than *X* was observed. In addition to these extra variables, leukocyte-leukocyte interaction count (number of times leukocytes come into close proximity or interact with each other), the path length (total distance travelled by a leukocyte), the dwell time (amount of time a leukocyte spends within a specific region of interest), and shape parameters (changes in leukocyte shape, such as elongation or circularity, during movement) might be invaluable parameters for reliable evaluation and modelling of the leukocyte-endothelium interaction type. However, these additional parameters are, in some cases, difficult to obtain or tedious to calculate, and in other cases, impossible to determine under the selected experimental conditions. Therefore, it was decided to merely investigate those parameters described in Table 1 in the following classification studies. Although a broad set of hydrodynamic descriptors was initially explored to comprehensively characterize leukocyte dynamics, it is important to emphasize that this variable pool is not intended to be used in its entirety. Instead, it serves as an exploratory space from which a limited subset of biologically meaningful parameters is systematically selected using statistical filtering and supervised multivariate modeling.

### Linear discriminant analysis for classification studies using advanced hydrodynamic variables

ANOVA and the HSD Tukey tests were performed for all the newly proposed hydrodynamic parameters across the distinct groups of individuals (see Table S9). As can be seen throughout the obtained *p-*values (Table S10), several parameters (${{v}_{x}^{\mathrm{med}}, v}_{x}^{0-500}$, ${v}_{x}^{500-1000}\cdot {a}_{x}^{\mathrm{med}}$, ${a}_{x}^{\left(-5000\right)-0}, {v}_{x}^{0-500}\cdot {v}_{x}^{500-1000}$, ${v}_{x}^{0-500}\cdot {v}_{x}^{1000-1500}$, ${v}_{x}^{500-1000}\cdot {v}_{x}^{1000-1500}$, ${N}_{{v}_{x}}^{0-500}$, ${N}_{{v}_{x}}^{500-1000}$, ${N}_{{v}_{x}}^{1000-1500}$, and ${N}_{{a}_{x}}^{5000-\infty }$) showed statistical differences between Healthy, T1D, and T2D groups, which demonstrate the potential of these parameters to construct LDA models with high discriminatory power. In addition, histograms of the newly proposed hydrodynamic parameters are shown in Fig. S3 to provide an intuitive visualization of their distribution across the different assayed groups. The distributions reveal clear shifts in central tendency and dispersion for several parameters, particularly between T1D and T2D, which are consistent with the statistically significant differences identified by ANOVA and Tukey’s HSD tests. These visual trends support the discriminatory capability of the selected novel parameters and are in full agreement with the quantitative results reported in Table S10. An interesting finding is that the heterogeneous distribution of leukocyte rolling velocities (${N}_{{v}_{x}}$) observed under basal conditions becomes markedly homogenized upon TNF-α activation, with the group-dependent differences largely disappearing. This suggests that inflammatory activation overrides the intrinsic variability present in the non-activated state, leading to more uniform leukocyte dynamics across Healthy, T1D, and T2D individuals.

**Fig. 3:**
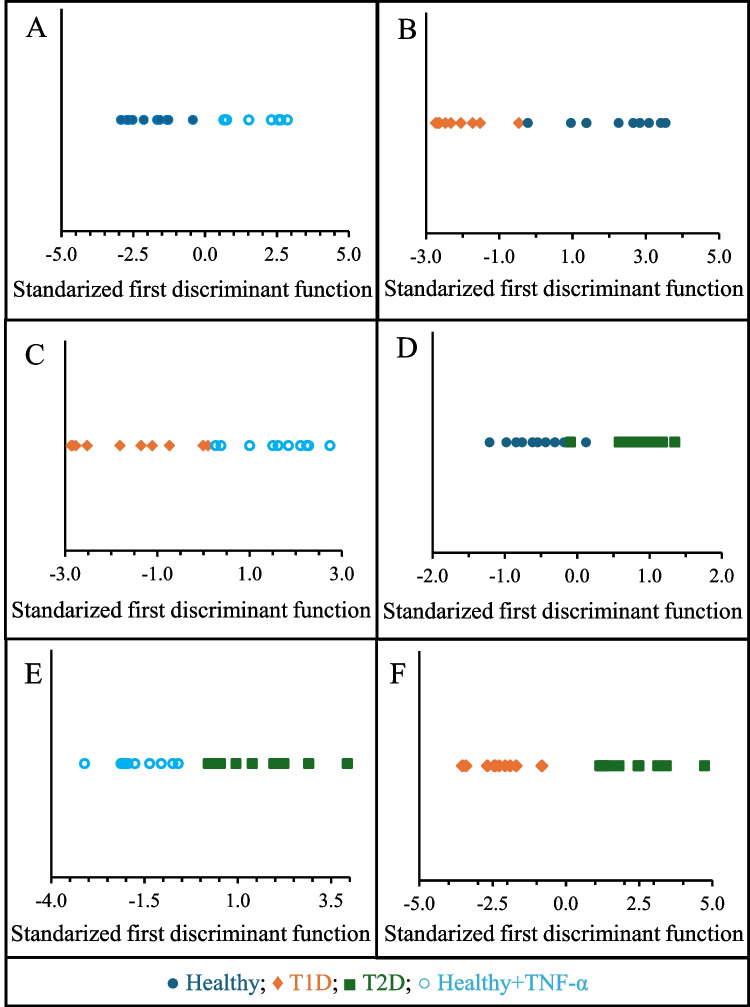
Different LDA plots constructed in 1 vs. 1 comparison: Healthy vs. Healthy+TNF-α (**A**), Healthy vs. T1D (**B**), Healthy+TNF-α vs. T1D (**C**), Healthy vs. T2D (**D**), Healthy+TNF-α vs. T2D (**E**), and T1D vs. T2D (**F**)

To further explore the multivariate structure underlying the advanced hydrodynamic parameters, PCA was performed on the standardized dataset as an unsupervised exploratory approach. PCA was not intended as a classification method, but rather to examine the intrinsic dimensionality of the flow-based variables and to assess potential redundancy among them. The PCA revealed that variance was progressively distributed across several components, with the first principal component explaining 31.6% of the total variance and the first two components together accounting for 46.3%. As illustrated by the scree plot (Fig. S4), eigenvalues exhibited a gradual decay without a dominant single component, indicating that leukocyte-endothelium interactions are, as expected, governed by a multidimensional dynamic behavior rather than by a single prevailing feature. Inspection of the component loadings showed that the first components were mainly driven by global descriptors of leukocyte motion, including average and median velocity, acceleration-related metrics, and variability measures, reflecting the overall dynamic activity. Subsequent components captured complementary information associated with intermediate velocity and acceleration ranges, interaction product variables, and ratios thereof, all reflecting rolling and motion heterogeneity. The varimax rotation further revealed a clear organization of variables into coherent functional domains, such as velocity-, acceleration-, interaction-, and count-related descriptors (Table S11), supporting their biological relevance and structured interdependence rather than an arbitrary grouping. Importantly, the projection of individual samples onto the PCA score space revealed substantial overlap among Healthy, T1D, and T2D groups, together with an increased dispersion in the diabetic groups. These observations indicate that group differences are not driven by a single dominant axis but emerge from the combined contribution of multiple complementary descriptors. Overall, PCA confirmed that the novel hydrodynamic variables contain structured and complementary information, while highlighting that robust discrimination between Healthy, T1D, and T2D groups requires supervised multivariate methods capable of integrating multiple descriptors simultaneously. Hence, utilizing the parameters outlined in Table 1 and the dataset showed in Table S9, LDA is again leveraged as a machine learning tool for discerning patterns and relationships within a multi-dimensional space. This approach enabled more precise differentiation between healthy subjects and T1D or T2D patients, between the two diabetes types, and in HUVECs activated or not with TNF-α. Thus, the relevance of the distinct variables chosen in every case study might be ascertained so as to ensure that the predictive models are rugged and accurate. To this end, six different binary LDA models were first constructed: Healthy vs. Healthy+TNF-α, Healthy vs. T1D, Healthy+TNF-α vs. T1D, Healthy vs. T2D, Healthy+TNF-α vs. T2D, and T1D vs. T2D. The results of these models and the corresponding graphical representations are shown in Table 2 and Fig. 3, respectively.

**Table 2:** LDA models with high classification accuracy for binary data comparison using advanced hydrodynamic variables

Model	Figure plot correspondence	Introduced variables (standardized coefficient)	VIF	*λ*_Wilks_	Classification accuracy	
Healthy vs. Healthy+TNF-α	Figure 3A	${v}_{x}^{500-1000}\times {v}_{x}^{1000-1500}$(1.000)	1.000	0.206	Original	Cross-validation
					Healthy: 90% Healthy+TNF-α: 100%	Healthy: 90% Healthy+TNF-α: 100%
Healthy vs. T1D	Figure 3B	$v_x^\mathrm{med}\times a_x^\mathrm{med}$(0.240) ${a}_{x}^{(-\infty )-(-5000)}\times {a}_{x}^{0-5000}$(−0.196) ${N}_{{v}_{x}}^{500-1000}$(1.002)	1.035 1.056 1.061	0.166	Original	Cross-validation
					Healthy: 90% T1D: 100%	Healthy: 90% T1D: 100%
Healthy+TNF-α vs. T1D	Figure 3C	${v}_{x}^{\mathrm{med}}$(−0.453) ${v}_{x}^{500-1000}\times {v}_{x}^{1000-1500}$(1.038)	1.009 1.009	0.261	Original	Cross-validation
					Healthy+TNF-α: 100% T1D: 90%	Healthy+TNF-α: 100% T1D: 80%
Healthy vs. T2D	Figure 3D	${R}_{\mathrm{ave}}^{+}/{R}_{\mathrm{ave}}^{-}$(0.862) ${v}_{x}^{0-500}\times {v}_{x}^{1000-1500}$(0.914) ${a}_{x}^{(-5000)-0}\times {a}_{x}^{5000-\infty }$(−0.341)	1.18 1.32 1.27	0.180	Original	Cross-validation
					Healthy: 100% T2D: 100%	Healthy: 90% T2D: 90%
Healthy+TNF-α vs. T2D	Figure 3E	${a}_{x}^{\left(-5000\right)-0}$(0.874) ${v}_{x}^{500-1000}\times {v}_{x}^{1000-1500}$(−0.993)	1.038 1.038	0.242	Original	Cross-validation
					Healthy+TNF-α: 100% T2D: 100%	Healthy+TNF-α: 100% T2D: 100%
T1D vs. T2D	Figure 3F	${v}_{x}^{0-500}\times {v}_{x}^{500-1000}$(1.001) ${R}_{\mathrm{ave}}^{+}/{R}_{\mathrm{ave}}^{-}$(0.952) $v_x^\mathrm{ave}$(−0.499)	1.002 1.161 1.002	0.162	Original	Cross-validation
					T1D: 100% T2D: 100%	T1D: 100% T2D: 100%

In the comparison between Healthy and Healthy+TNF-α individuals (Fig. 3A), only one variable ${v}_{x}^{500-1000}\times {v}_{x}^{1000-1500}$ was necessary in as much as a *λ*_*w*_ as low as 0.206 was obtained along with an excellent classification accuracy (95%) for both original (complete dataset) and cross-validation datasets (complete dataset except the individual analyzed). This variable captures the interaction between mid-range and high-range velocities of leukocytes, which are notably distinct in TNF-α activated condition. Specifically, it is generally expected that mid-range velocities increase relative to high-range velocities in inflammatory conditions, such as those with TNF-α activation, due to stronger interactions of leukocytes with the endothelium. Therefore, the selected variable, which combines these aspects into a single measure, effectively differentiates between Healthy and inflammatory conditions by highlighting the relevance of separating leukocytes with varied velocity ranges. This initial result is crucial in demonstrating that variables beyond the conventional parameters can effectively differentiate between two distinct endothelial conditions.

When comparing Healthy individuals to those with T1D (Fig. 3B), three key variables were identified: ${v}_x^{med}\times a_x^{med},a_x^{\left(-\infty\right)-\left(-5000\right)}\times a_x^{0-5000}$, and $N_{{\mathrm v}_{\mathrm x}}^{500-1000}$. These variables achieved a Wilks’ lambda of 0.166 and x excellent classification accuracy, even for the cross-validation (95%). The product of median velocity with median acceleration provides a comprehensive view of how leukocyte speed relates to their acceleration. In patients with T1D, changes of this product might reflect altered leukocyte dynamics, such as increased or decreased responsiveness in terms of speed and acceleration, indicating underlying inflammatory processes. Specifically, in a high-inflammatory condition, both velocity and acceleration might decrease, leading to a significant reduction in their product. Hence, low values could be interpreted as indicative of greater affinity of the leukocytes, while high values may reflect free-flowing leukocytes with negligible interaction with the endothelium. The specific acceleration ranges highlight how T1D affects leukocyte movement, particularly in how leukocytes transition between different acceleration conditions. The count of leukocytes in the 500–1000 velocity range reflects the number of leukocytes moving at these mid-range velocities, which is indicative of inflammatory responses. These variables effectively capture the complex inflammatory responses characteristic of T1D.

For the comparison between Healthy+TNF-α and T1D individuals (Fig. 3C), the variables ${v}_{x}^{\mathrm{med}}$ and ${v}_{x}^{500-1000}\times {v}_{x}^{1000-1500}$ were critical, achieving a Wilks’ lambda of 0.261 and again an excellent classification accuracy for complete dataset (95%) and for cross-validation (90%). The median velocity and the interaction between mid-range and high-range velocities provide significant insights into the differing leukocyte dynamics between TNF-α activation and T1D. Unlike the previous two studies, in which comparisons involved healthy versus actual inflammatory conditions, both TNF-α activation and T1D are related to inflammatory conditions. As a result, different variables are necessary to explain the differences in leukocyte behavior. Despite ${v}_{x}^{\mathrm{med}}$ may vary between these conditions, the wide range of diabetes levels suggests that additional variables are needed for more reliable differentiation. In fact, the interaction between mid-range and high-range velocities is herein useful to discriminate leukocyte movement patterns for both inflammatory conditions. These variables underscore the fundamental differences in leukocyte behavior, even when comparing inflammatory conditions.

The discrimination between Healthy individuals and those with T2D (Fig. 3D) based on the leukocyte-endothelium interaction was also feasible; however, different variables were needed. In this instance, the variables ${R}_{\mathrm{ave}}^{+}/{R}_{\mathrm{ave}}^{-}$, ${v}_{x}^{0-500}\times {v}_{x}^{1000-1500}$, and ${a}_{x}^{(-5000)-0}\times {a}_{x}^{5000-\infty }$ were pivotal, achieving a Wilks’ lambda of 0.180 and 100% classification accuracy and 90% for cross-validation. This signals that the differences between the healthy condition and the inflammatory condition in T2D are characterized by interactions distinct from those in T1D. In this context, the balance between positive and negative rolling might indicate specific changes in the endothelial environment and leukocyte interactions. Generally, lower values of this ratio are indicative of stronger inflammatory events. Similarly, as observed in previous cases, the product ${v}_{x}^{0-1000}\times {v}_{x}^{1000-1500}$ demonstrated distinct dynamic movement patterns of the leukocyte population for T2D compared to healthy conditions. For the acceleration product, ${a}_{x}^{(-5000)-0}\times {a}_{x}^{5000-\infty }$, a similar behavior to the velocity product is anticipated; the interaction of leukocytes with the endothelium will lead to different accelerations depending on the inflammatory condition. Thus, these variables effectively reflect the subtle alterations in leukocyte behavior indicative of T2D.

The comparison between Healthy+TNF-α and T2D individuals (Fig. 3E) identified ${a}_{x}^{\left(-5000\right)-0}$ and ${v}_{x}^{500-1000}\times {v}_{x}^{1000-1500}$ as critical variables, resulting in a Wilks’ lambda of 0.242 and 100% classification accuracy. The relevance of leukocyte dynamics within the moderate negative acceleration range $\left({a}_{x}^{\left(-5000\right)-0}\right)$ may reflect sustained rolling and progressive strengthening of leukocyte-endothelium interactions rather than abrupt arrest. This behavior is consistent with a scenario of endothelial activation, as observed in T2D, in which leukocytes experience repeated deceleration events due to prolonged interaction with adhesion molecules, without transitioning rapidly to firm adhesion. Together with the product interaction between mid-range and high-range velocities, this variable captures a distinctive dynamic signature that enables accurate discrimination between TNF-α-induced activation and T2D. It is important to note that the velocity product of the mid-range and high-range velocities is a common variable for the comparison of Healthy+TNF-α against both T1D and T2D inflammatory conditions. This might however complicate differentiating the two groups with diabetes.

Interestingly, the LDA binary model with the new flow parameters showed a substantial improvement in discriminating between T1D and T2D patients (Fig. 3F). This model achieved a low *λ*_*w*_ (0.162), indicating good group separation, and yielded excellent classification in the training set (100% for both T1D and T2D). More importantly, cross-validation accuracy also achieved 100% for T1D and for T2D. In contrast to the poorer performance observed with classical parameters (*λ*_*w*_ = 0.582; 80 and 75% classification for the complete dataset and cross-validation, respectively), these results demonstrate that the newly proposed flow parameters capture subtle dynamic differences in cell behavior that are not reflected by the classical metrics. Therefore, these advanced descriptors provide more biologically informative features, improving LDA model robustness and offering greater potential for clinical discrimination between diabetes subtypes, even with training sets containing a limited number of individuals.

In addition to the excellent classification performance, the ruggedness of the binary LDA models was supported by the evaluation of multicollinearity. Variance inflation factor (VIF) values were consistently close to unity for all selected variables in the models shown in Table 2, indicating that the descriptors capture complementary aspects of leukocyte dynamics rather than redundant information. The low degree of collinearity reinforces the interpretability of the LDA models and supports the biological relevance of each hydrodynamic parameter included.

**Fig. 4:**
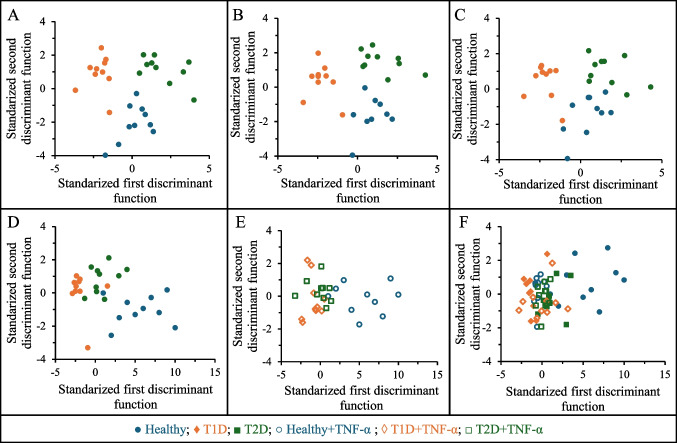
Ternary LDA plots aimed at discriminating: Healthy vs. T1D vs. T2D using six (**A**), five (**B**), four (**C**), and three (**D**) variables; Healthy+TNF-α vs. T1D+TNF-α vs. T2D+TNF-α (**E**), and Healthy vs. Healthy+TNF-α vs. T1D vs. T1D+TNF-α vs. T2D vs. T2D+TNF-α (**F**)

The above preliminary studies of one against another group highlight the potential of expanding these novel hydrodynamic variables to develop more complex ternary models that can differentiate simultaneously healthy individuals against those with inflammatory conditions derived from T1D, and also those from T2D (all with or without TNF-α). Consequently, Fig. 4 and Table 3 illustrate various ternary (or larger) LDA models with those variables featuring high discriminatory power.

**Table 3:** Ternary LDA models constructed for the comparison of Healthy vs. T1D vs. T2D using six, five, and three variables; Healthy+TNF-α vs. T1D+TNF-α vs. T2D+TNF-α, Healthy/Healthy+TNF-α vs. T1D/T1D+TNF-*α vs.* T2D/T2D+TNF-α and Healthy vs. Healthy+TNF-α vs. T1D vs. T1D+TNF-α vs. T2D vs. T2D+TNF-α

Model	Figure plot correspondence	Introduced variables (standarized coeficients: F1; F2: F3)	VIF	λ_Wilks_	Classification results	
Healthy vs. T1D vs*.* T2D	Figure 4A	${N}_{{v}_{x}}^{500-1000}$(0.527; −0.77) ${N}_{{v}_{x}}^{1000-1500}$ (−0.264; 0.943) ${v}_{x}^{0-500}\times {v}_{x}^{500-1000}$ (0.602; 0.344) ${R}_{\mathrm{ave}}^{+}/{R}_{\mathrm{ave}}^{-}$ (0.538; 0.684) ${v}_{x}^{\mathrm{ave}}$ (−0.062; −0.821) ${a}_{x}^{\left(-\infty \right)-(-5000)}$ (−0.307; −0.256)	2.92 2.43 1.58 1.18 1.18 1.07	0.298	Original	Cross-validation
					Healthy: 100% T1D: 90% T2D: 100%	Healthy: 90% T1D: 90% T2D: 90%
Healthy vs. T1D vs. T2D	Figure 4B	${N}_{{v}_{x}}^{500-1000}$(0.556; 0.105) ${N}_{{v}_{x}}^{1000-1500}$ (−0.5070; 0.834) ${v}_{x}^{0-500}\times {v}_{x}^{500-1000}$ (0.4689; 0.459) ${R}_{\mathrm{ave}}^{+}/{R}_{\mathrm{ave}}^{-}$ (0.324; 0.780) ${v}_{x}^{\mathrm{ave}}$ (0.155; −0.811)	2.85 2.44 1.58 1.18 1.18	0.316	Original	Cross-validation
					Healthy: 100% T1D: 90% T2D: 100%	Healthy: 90% T1D: 90% T2D: 100%
Healthy vs. T1D vs. T2D	Figure 4C	${N}_{{v}_{x}}^{500-1000}$(0.777; −0.259) ${N}_{{v}_{x}}^{1000-1500}$ (−0.539; 0.734) ${v}_{x}^{0-500}\times {v}_{x}^{500-1000}$ (0.409; 0.552) ${R}_{\mathrm{ave}}^{+}/{R}_{\mathrm{ave}}^{-}$ (0.325; 0.344)	2.76 2.27 1.43 1.12	0.451	Original	Cross-validation
					Healthy: 90% T1D: 90% T2D: 100%	Healthy: 90% T1D: 90% T2D: 100%
Healthy vs. T1D vs. T2D	Figure 4D	${N}_{{v}_{x}}^{500-1000}$(0.711; 0.464) ${N}_{{v}_{x}}^{1000-1500}$ (−0.437; 0.821) ${R}_{\mathrm{ave}}^{+}/{R}_{\mathrm{ave}}^{-}$ (0.245; 0.702)	2.04 1.90 1.11	0.588	Original	Cross-validation
					Healthy: 80% T1D: 90% T2D: 90%	Healthy: 70% T1D: 90% T2D: 80%
Healthy+TNF-α vs. T1D+TNF-α vs. T2D+TNF-α	Figure 4E	${v}_{x}^{500-1000}$(0.543; −0.919) ${v}_{x}^{1500-\infty }$ (0.434; 0.343) ${N}_{{a}_{x}}^{5000-\infty }$ (0.496; 0.718)	1.49 1.17 1.48	0.934	Original	Cross-validation
					Healthy+TNF-α: 70% T1D+TNF-α: 70% T2D+TNF-α: 50%	Healthy+TNF-α: 60% T1D+TNF-α: 50% T2D+TNF-α: 50%
Healthy vs. Healthy+TNF-α vs. T1D vs. T2D vs. T1D+TNF-α vs. T2D+TNF-α	Figure 4F	${N}_{{v}_{x}}^{500-1000}$(0.855; 0.356; −0.376) ${R}_{\mathrm{ave}}^{+}/{R}_{\mathrm{ave}}^{-}$ (−0.108; 0.770; 0.629) ${v}_{x}^{\mathrm{ave}}$ (0.364; −0.187; 0.912)	1.01 1.12 1.12	0.936	Original	Cross-validation
					Healthy: 50% Healthy+TNF-α: 80% T1D: 80% T1D+TNF-α: 40% T2D: 30% T2D+TNF-α: 50%	Healthy: 40% Healthy+TNF-α: 70% T1D: 60% T1D+TNF-α: 20% T2D: 20% T2D+TNF-α: 20%

To develop a ternary LDA model capable of differentiating between Healthy, T1D, and T2D individuals simultaneously, four different sets of variables were tested. The first model (Fig. 4A) utilized six variables (${N}_{{v}_{x}}^{500-1000}$; ${N}_{{v}_{x}}^{1000-1500}$; ${v}_{x}^{0-500}\times {v}_{x}^{500-1000}$; ${R}_{\mathrm{ave}}^{+}/{R}_{\mathrm{ave}}^{-}$; ${v}_{x}^{\mathrm{ave}}$; and ${a}_{x}^{\left(-\infty \right)-(-5000)}$) and achieved a Wilks’ lambda of 0.298. This model demonstrated an excellent classification accuracy (96.7%) and good cross-validation classification accuracy (90%). However, it misclassified few individuals when cross-validation is used, indicating the potential for false positives and false negatives. These errors might have significant clinical implications, such as failing to provide appropriate medication to a patient with an inflammatory condition like diabetes. Although cross-validation revealed the misclassification of a small number of individuals, this ternary model still demonstrates a remarkably high discriminatory capacity given the limited sample size and the intrinsic biological heterogeneity of diabetes. Importantly, the overall classification performance remained robust, with high accuracy for all three groups, indicating that the selected hydrodynamic variables capture biologically meaningful patterns of leukocyte-endothelium interactions. Hence, this observation underscores the sensitivity of the model, which is capable of detecting subtle immune-related changes even at early stages of potential pathophysiological development. Some of the variables used in this LDA model are akin to those found relevant in the 1 vs. 1 models, such as ${N}_{{v}_{x}}^{500-1000}$ from Healthy vs. T1D, ${v}_{x}^{0-500}\times {v}_{x}^{1000-1500}$ and ${R}_{\mathrm{ave}}^{+}/{R}_{\mathrm{ave}}^{-}$ from the Healthy vs. T2D and T1D vs. T2D and ${v}_{x}^{\mathrm{ave}}$ from T1D vs. T2D models. This model required six variables to adequately predict patient status, which is partially a large model, although is still not close to the acceptable limit of variables (one-third of the samples, which is 10 in this case). Hence, three additional models were constructed with just five (Fig. 4B), four (Fig. 4C), and three (Fig. 4D) variables. The five-variable model excluded ${a}_{x}^{\left(-\infty \right)-(-5000)}$, the four-variable models excluded ${a}_{x}^{\left(-\infty \right)-(-5000)}$ and ${v}_{x}^{\mathrm{ave}}$, and the three-variable model excluded ${a}_{x}^{\left(-\infty \right)-(-5000)}$, ${v}_{x}^{\mathrm{ave}}$, and ${v}_{x}^{0-500}\times {v}_{x}^{500-1000}$. As expected, the four-variable and the three-variable models showed lower prediction capabilities for cross-validation (90–100% and 70–90%, respectively) and a larger Wilks’ lambdas of 0.451 and 0.588, respectively. Surprisingly, the five-variable model (Fig. 4B) exhibited strong prediction capabilities for cross-validation (90–100%) and a good Wilks’ lambda of 0.316. Therefore, this variable combination is a more acceptable prediction model for Healthy, T1D, and T2D individuals than the one with six variables.

To assess the overall prediction capability, two additional ternary models including data of TNF-α activated endothelium were tested: Healthy+TNF-α vs. T1D+TNF-α vs. T2D+TNF-α (Fig. 4E). The LDA identified a model with three variables, ${v}_{x}^{500-1000}$, ${v}_{x}^{1500-\infty }$, and ${N}_{{a}_{x}}^{5000-\infty }$, resulting in a moderate classification accuracy (50–60%) but with poor Wilks’ lambda value (0.934). This suggests that TNF-α activation homogenizes the inflammation condition, failing to capture the actual changes in leukocyte dynamics among Healthy, T1D, and T2D conditions. These results are in agreement with the change in the ${N}_{{v}_{x}}$ behavior after TNF-α activation (Fig. S3). The latter model showed low accuracy in predicting T2D individuals, leading to false negatives and lack of discrimination against T1D individuals, rendering the LDA classification method less suitable for practical applications. Next, the evaluation of all groups simultaneously in one model was attempted (Fig. 4F). However, this model exhibited low cross-validation prediction accuracy (20–70%), indicating poor overall performance. The robustness of the ternary LDA models was also evaluated by assessing multicollinearity among the selected variables. VIF values remained low and close to unity across all models (Table 3), even in those including a higher number of descriptors, indicating that the retained variables provide complementary rather than redundant information. Together with the parsimonious variable selection strategy, this supports the stability and interpretability of the ternary classification models.

Overall, the LDA highlights that a consistent subset of novel flow-based variables repeatedly emerges across multiple classification models, underscoring their central role in discriminating healthy individuals from those with T1D or T2D, as well as from TNF-α–activated endothelial conditions. Rather than being driven by a single descriptor, model performance relies on complementary information provided by global velocity metrics, velocity distribution across mid-range regimes, interaction terms between adjacent velocity ranges, and rolling balance indicators. In particular, the average leukocyte velocity $\left({v}_{x}^{\mathrm{ave}}\right)$, the number of leukocytes within intermediate velocity ranges $\left(N_{\left(v_x\right)}^{500-1000},N_{\left(v_x\right)}^{1000-1500}\right)$, the interaction between low- and mid-range velocities $\left(v_x^{0-500}\times v_x^{500-1000}\right)$, and the ratio of positive to negative rolling events $\left({R}_{\mathrm{ave}}^{+}/{R}_{\mathrm{ave}}^{-}\right)$ collectively capture distinct yet complementary aspects of leukocyte-endothelium dynamics. Together, these recurrent variables reflect changes in leukocyte motility, rolling events, and population heterogeneity, reinforcing the importance of incorporating advanced hydrodynamic descriptors to improve the ruggedness and predictive power of inflammatory classification models. Next, the 30 individuals studied in this work were randomly split into two groups: the training group (individuals 3738, 3744, 3812, 3816, 3858, 3860, 4616, 3776, 3737, 3761, 3772, 3775, 3876, 4787, 3748, 3777, 3809, 3811, 3821, 3836, and 3852), which included four individuals from each health condition (Healthy, T1D, and T2D), and the testing group (individuals 3679, 3741, 3861, 3760, 3774, 3878, 3755, 3835, and 3840). The training group was used to build the LDA model based on the five variables previously selected in Fig. 3B. The testing group was then used to validate the model. The results showed 100% correct classification for all the testing individuals. This again corroborates the prediction capability of the newly proposed flow variables and the developed models in predicting diabetes by analyzing the dynamic flow-through interactions between the leukocytes and the endothelium tissue. Notably, despite the large initial number of candidate descriptors, the final classification models consistently relied on a small number of variables (3–6), which were repeatedly selected across different comparisons. Despite the relatively limited cohort size per group (*n* = 10), the variables consistently demonstrated strong discriminatory capacity across independent classification scenarios, supporting biological interpretability and potential translational relevance, as discussed in the following.

Previous studies have reported that both T1D and T2D are associated with altered leukocyte-endothelium interactions, although the mechanisms may differ. In T2D, increased rolling flux, reduced rolling velocity, and enhanced firm adhesion are particularly related to poor glycaemic control [18]. Moreover, changes in relevant leukocyte CAMs, such as impaired function of PSGL-1 [27] and higher expression of CD11b and CD66b, have been described in T2D [28], which might be related to endothelial damage and cardiovascular risk. In contrast, other alterations have been found in selectins and integrins in T1D. For instance, some authors reported elevated soluble l-selectin levels, associated with autoimmunity and potential destruction of beta cells, and upregulation of β2 integrins such as Mac-1 (CD11b) in T1D, which may be relevant in the initiation of insulitis that facilitates leukocyte adhesion [29]. These findings suggest that T1D involves acute, immune-driven inflammation, whereas T2D reflects chronic low-grade metabolic inflammation, highlighting the need for refined quantitative tools to distinguish these phenotypes. Therefore, the proposed new hydrodynamic parameters are capable of finding these differences, allowing the adequate classification of the different inflammatory behaviors.

To further assess the potential relationship between circulating biomarkers and the leukocyte dynamic behavior, Pearson (Table S12) and Spearman (Table S13) correlation analyses (990 comparisons each) were performed between the biochemical parameters and the complete set of the newly proposed hydrodynamic variables. Overall, the correlation coefficients were predominantly weak to moderate, indicating that leukocyte-endothelium dynamics partially reflect the underlying inflammatory events but are not directly explained by individual biomarkers. Across all biomarker-hydrodynamic comparisons, Pearson absolute correlation coefficients exhibited a median value of approximately 0.15, with fewer than 25% of correlations exceeding 0.23. In comparison, Spearman coefficients showed lower overall magnitudes, with median absolute values around 0.13 and with only a few cases of higher correlations, indicating that the associations between biochemical markers and hydrodynamic descriptors are predominantly linear rather than purely monotonic. This observation further supports the suitability of parametric multivariate approaches, such as linear discriminant analysis, for integrating hydrodynamic variables into classification models.

In Pearson analysis, despite the overall moderate correlation, coherent and biologically meaningful patterns were obtained across groups of variables. In particular, several interleukins (including IL-1β, IL-2, IL-5, IL-8, and IL-13) showed preferential associations with interaction terms involving low- and mid-range velocity regimes, such as the products between leukocyte velocities in the 0–500 and 500–1000 μm/s ranges. These relationships suggest that cytokine-mediated inflammatory signaling is primarily linked to early rolling and transitional leukocyte dynamics, in which frequent velocity fluctuations and short-lived adhesive interactions dominate. In contrast, MPO displayed stronger associations with variables related to counting variables of intermediate velocity ranges and some acceleration ranges (Table S12), consistent with its role as a marker of neutrophil activation and sustained endothelial engagement. This pattern supports the knowledge that MPO levels reflect more established inflammatory states that are characterized by prolonged leukocyte-endothelium interactions rather than transient rolling events. In contrast, the variables selected by the LDA model predominantly captured sustained leukocyte-endothelium interactions, supporting the interpretation that MPO levels reflect established inflammatory states rather than transient rolling dynamics. The strongest Pearson correlation reached values close to *r* ≈ 0.70 and corresponded to the relationship between GM-CSF levels and the ratio of average leukocyte velocity to average acceleration. GM-CSF is known to induce leukocyte priming and to modulate integrin-mediated interactions, promoting sustained but regulated endothelial interactions rather than abrupt arrest. Accordingly, higher GM-CSF concentrations may be associated with leukocyte trajectories characterized by relatively stable velocities and reduced fluctuations, leading to a stronger linear relationship between velocity and acceleration.

In the Spearman analysis, as noted above, overall correlation magnitudes were lower than those observed with Pearson coefficients, indicating weaker monotonic associations between circulating biomarkers and hydrodynamic variables. Nevertheless, consistent qualitative patterns could still be identified. Several interleukins, particularly IL-1β, IL-4, IL-5, and IL-13, exhibited preferential monotonic relationships with interaction terms involving low- and mid-range velocity regimes, especially products between leukocyte velocities in the 500–1000 and 1000–1500 μm/s ranges, with correlation coefficients reaching moderate values (*ρ* ≈ − 0.60 to − 0.65). These associations suggest that cytokine-driven inflammatory activity is broadly linked to shifts in leukocyte velocity distributions rather than to sharply defined dynamic states. In contrast, MPO showed moderate monotonic associations with descriptors related to intermediate counting velocity regimes (*ρ* ≈ − 0.47) and some acceleration ranges (*ρ* ≈ − 0.45), with enhanced strength compared to Pearson correlations. This behavior is consistent with MPO reflecting cumulative neutrophil activation and sustained endothelial interactions, rendering gradual changes in leukocyte dynamics rather than abrupt transitions. Importantly, the hydrodynamic descriptors associated with the highest Spearman coefficients largely overlapped with those identified in the Pearson analysis, indicating that both linear and monotonic approaches converge in highlighting similar dynamic regimes despite differences in the correlation magnitudes.

**Fig. 5:**
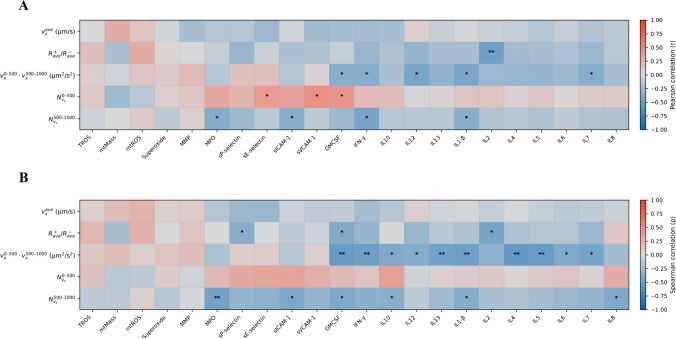
Heat map of Pearson (**A**) and Spearman (**B**) coefficients for the comparison between circulating biomarkers and the most relevant newly introduced hydrodynamic variables in the LDA models. * and ** indicate *p*-values lower than 0.05 and 0.01, respectively

Importantly, the most frequently retained hydrodynamic variables in the LDA classification models also exhibited biologically interpretable associations (Fig. 5) with multiple circulating biomarkers, despite not yielding the highest univariate correlation coefficients. The average leukocyte velocity $\left({v}_{x}^{\mathrm{ave}}\right)$ showed moderate negative Pearson and Spearman correlations with inflammatory markers, indicating that increasing the inflammatory burden is associated with a global reduction in leukocyte motility under flow conditions. The rolling balance parameter $\left({\mathrm{R}}_{\mathrm{ave}}^{+}/{\mathrm{R}}_{\mathrm{ave}}^{-}\right)$ exhibited predominantly negative correlations with several circulating biomarkers in both Pearson and Spearman analyses. In the Pearson analysis, the strongest negative associations were observed with IL-2 (*r* =  − 0.49), IL-12 (*r* =  − 0.31), IFN-γ (*r* =  − 0.30), and sP-selectin (*r* =  − 0.30), together with moderate correlations with IL-8 (*r* =  − 0.28) and IL-1β (*r* =  − 0.22). Consistently, Spearman analysis revealed negative monotonic relationships with IL-2 (*ρ* =  − 0.45), GM-CSF (*ρ* =  − 0.40), sP-selectin (*ρ* =  − 0.37), IFN-γ (*ρ* =  − 0.31), and IL-12 (*ρ* =  − 0.30). The recurrence of IL-2, IFN-γ, and IL-12 across both correlation metrics highlights a robust association between Th1-type immune activation and rolling events. Functionally, these patterns suggest that elevated immune activation promotes a predominance of deceleration and reduced rolling fluctuations, reflecting enhanced adhesive effects during leukocyte-endothelium interactions. The interaction term between low- and intermediate-range leukocyte velocities $\left(v_x^{0-500}\times v_x^{500-1000}\right)$ showed consistent and predominantly negative associations with multiple circulating cytokines in both Pearson and Spearman analyses. In the Pearson analysis, moderate negative correlations were observed with several pro-inflammatory mediators, including GM-CSF (*r* =  − 0.37), IFN-γ (*r* =  − 0.37), IL-12 (*r* =  − 0.44), IL-1β (*r* =  − 0.43), IL-6 (*r* =  − 0.38), and IL-13 (*r* =  − 0.33), indicating that increasing inflammatory signaling is associated with significant variations of low and intermediate velocity regimes. This trend was even more pronounced in the Spearman analysis, in which stronger monotonic negative correlations were detected with GM-CSF (*ρ* =  − 0.53), IFN-γ (*ρ* =  − 0.49), IL-1β (*ρ* =  − 0.49), IL-4 (*ρ* =  − 0.54), IL-5 (*ρ* =  − 0.51), IL-13 (*ρ* =  − 0.47), and IL-7 (*ρ* =  − 0.45). The systematic nature of these negative associations suggests that cytokine-driven inflammatory activation disrupts the coordinated transition between rolling and faster dynamic states, leading to a decoupling of leukocyte behavior across adjacent velocity regimes. Functionally, this reflects a shift toward either more stabilized rolling or more abrupt transitions, rather than smooth redistribution across dynamic states. Variables describing the distribution of leukocytes within specific velocity regimes showed complementary and highly structured associations with circulating biomarkers. The number of leukocytes within the lowest velocity range $\left(N_{v_x}^{0-500}\right)$ exhibited predominantly positive correlations with markers of endothelial activation and immune signaling in both Pearson and Spearman analyses. In particular, positive associations were observed with sE-selectin (*r* = 0.39; *ρ* = 0.32), sICAM-1 (*r* = 0.33; *ρ* = 0.32), svCAM-1 (*r* = 0.45; *ρ* = 0.26), GM-CSF (*r* = 0.42; *ρ* = 0.19), and MPO (*r* = 0.30; *ρ* = 0.20), indicating that increased inflammatory and endothelial activation is associated with a higher proportion of leukocytes populating very low velocity regimes that are characteristic of stabilized rolling or near-arrest states. In contrast, the number of leukocytes within intermediate velocity ranges $\left(N_{v_x}^{500-1000}\right)$ showed predominantly negative correlations with many of the same biomarkers. Pearson analysis revealed moderate negative associations with MPO (*r* =  − 0.39), IFN-γ (*r* =  − 0.45), sICAM-1 (*r* =  − 0.37), GM-CSF (*r* =  − 0.28), and IL-1β (*r* =  − 0.37), while Spearman analysis further strengthened these trends, particularly for MPO (*ρ* =  − 0.47), sICAM-1 (*ρ* =  − 0.42), GM-CSF (*ρ* =  − 0.41), and IL-8 (*ρ* =  − 0.41). Together, these opposite correlation patterns indicate a redistribution of leukocyte populations from intermediate toward lower velocity regimes as inflammatory activation increases. This coordinated shift underscores the sensitivity of velocity-range-based descriptors to elucidate endothelial activation and leukocyte priming and explains their recurrent selection in the classification models, despite their moderate univariate correlation magnitudes.

## Conclusions

This study presents the first direct comparative analysis of leukocyte-endothelium dynamics in clinical samples from patients with T1D and T2D using novel hydrodynamic parameters. Traditionally, leukocyte-endothelium interactions have been described using a narrow set of classical parameters: rolling velocity, rolling flux, and adhesion. While useful for capturing broad differences between healthy and inflammatory states, these metrics proved inadequate for reliable differentiation of T1D from T2D. The inability of these descriptors to capture the subtle, yet biologically meaningful, differences in cell behavior highlights the need for more sensitive analytical approaches. By extracting a broader set of hydrodynamic variables from high-resolution video recordings, including velocity, acceleration, and rolling variables, along with their standard deviation, interaction product, and count variables, the novel descriptors were able to capture higher-order features of leukocyte motion.

Supervised machine learning models based on LDA, combined with systematic variable selection and cross-validation strategies, demonstrated that only a limited and biologically interpretable subset of these novel descriptors is required for reliable discrimination between Healthy, T1D, and T2D individuals. LDA models built with the novel variables reached 100% classification accuracy between T1D and T2D and healthy individuals in the training set, with excellent cross-validation and external testing results. These findings should be interpreted within a proof-of-concept framework, underscoring both the ruggedness and the translational potential of the proposed approach, even with a limited sample size.

Among the new variables introduced, those based on the product of velocity and acceleration emerged as particularly informative. Reduced values of this metric in T1D reflect sustained endothelial contact and decreased leukocyte motility, consistent with acute immune-driven inflammation events. By contrast, T2D leukocytes displayed more heterogeneous behavior, characterized by increased adhesion and intermittent accelerations, aligning with chronic low-grade metabolic inflammation conditions. Equally important was the assessment of rolling event ratios, which quantify the balance between acceleration (detachment) and deceleration (adhesion) episodes. A predominance of deceleration events in T2D samples suggests persistent leukocyte-endothelium interactions, whereas T1D exhibited a different pattern indicative of transient but stronger immune activation. Thus, these metrics serve as functional biomarkers that directly map onto the pathophysiological mechanisms of each diabetes subtype.

Correlation analyses using both Pearson and Spearman coefficients revealed predominantly weak to moderate associations between circulating inflammatory biomarkers and the novel hydrodynamic descriptors. Rather than limiting biological interpretability, this partial correlation indicates that leukocyte dynamic behavior captures complementary, higher-order functional information that is not fully explained by individual molecular markers.

From the perspective of analytical chemistry, this work introduces an entirely new class of flow-based descriptors for cell-endothelium interactions. By leveraging digital video processing and supervised machine learning tools such as LDA, we have demonstrated how dynamic biological processes can be transformed into quantitative analytical variables with diagnostic potential. This methodological innovation expands the analytical toolbox available for studying inflammation and vascular biology, offering a platform for the development of reproducible, high-throughput assays. Furthermore, the demonstration that only a limited number of novel descriptors is sufficient to achieve robust classification underscores the efficiency of this approach.

Although these findings should not yet be interpreted as directly applicable to clinical decision-making, they highlight the potential of leukocyte hydrodynamic profiling as a functional tool to characterize distinct inflammatory phenotypes associated with T1D and T2D. Such discrimination is crucial, given the fact that both forms of diabetes lead to increased vascular morbidity but through distinct immunological and metabolic pathways. In the future, hydrodynamic descriptors may contribute to the development of functional assays aimed at monitoring inflammatory status under controlled flow conditions, pending validation in larger and independent cohorts.

Future research is therefore required to evaluate the practicality of these hydrodynamic parameters in therapeutic interventions, including glucose-lowering strategies, lifestyle modifications, and targeted anti-inflammatory agents, within appropriately powered and longitudinal study designs. The monitoring of the dynamic leukocyte behavior may represent an invaluable supplement to the clinical field, pending validation in larger and longitudinal clinical studies. Overall, this work establishes a proof-of-concept framework in which dynamic leukocyte-endothelium interactions under flow conditions are proposed as functional biomarkers, laying the groundwork for future translational and precision medicine–oriented studies.

## Supplementary information

Below is the link to the electronic supplementary material.

ESM 1 (PDF 778 KB)

ESM 2 (XLSX 88.6 KB)

## Data Availability

The data generated and analyzed during the current study, if it is not included in the manuscript, is available from the corresponding author upon request.
