# Supplementary material for: Reliable discrimination of type 1 and type 2 diabetes by flow-through leukocyte-endothelium interactions exploiting advanced hydrodynamic detection parameters
Source: Anal Bioanal Chem. 2026 Feb 16;418(8):2481–502. doi: 10.1007/s00216-026-06377-6 (PMC13065618; doi:10.1007/s00216-026-06377-6)
Supplement: Supplementary file 1 — (PDF 778 KB) [file 216_2026_6377_MOESM1_ESM.pdf]

SUPPLEMENTARY INFORMATION

Reliable discrimination of type 1 and type 2 diabetes by flow-through leukocyte-endothelium interactions exploiting advanced hydrodynamic detection parameters

Jonathan Hermenejildo[1#], Sandra López-Doménech[1#], María Pelechá-Salvador[1], Carlos Morillas[1], Milagros Rocha[1,2*], Manuel Miró[3], Enrique Javier Carrasco-Correa[4*]

[1] Department of Endocrinology and Nutrition, University Hospital Doctor Peset, Foundation for the Promotion of Health and Biomedical Research in the Valencian Region (FISABIO), 46017 Valencia, Spain.

[2] CIBEREHD (Centro de Investigación Biomédica en Red de Enfermedades Hepáticas y Digestivas), 28029, Madrid, Spain.

[3]FI-TRACE Group, Department of Chemistry, University of the Balearic Islands, Carretera de Valldemossa, km 7.5, Palma, 07122, Spain

[4]CLECEM group, Department of Analytical Chemistry, Faculty of Chemistry, University of Valencia, Av. Vicent Andrés Estellés, 19, 46100-Burjassot, València, Spain

# These authors have contributed equally

*Corresponding authors:

Milagros Rocha-Barajas

e-mail: milagros.rocha@fisabio.es

Dr. Enrique Javier Carrasco-Correa

e-mail: enrique.carrasco@uv.es

Pages: 23

Figures: 4

Tables: 13

Table S1. Anthropometric and biochemical parameters of the study population. *p*-values under 0.05 are highlighted in bold.

	Healthy	T1D	T2D	p-value	p-value adjusted by age
**n**	10	10	10	-	-
**Women (%)**	60	50	30	0.404	-
**Duration of diabetes (yr)**	-	18.0 ± 15.4	14.9 ± 2.8	0.950	-
**Age (yr)**	40.5 ± 8.6[a]	45.6 ± .9.8[a]	64.4 ± 4.2[b]	**<0.001**	-
**BMI (kg/m^2)**	25.3 ± 5.6	25.4 ± 3.9	28.9 ± 3.9	0.158	0.246
**Glucose (mg/dL)**	84 ± 9[a]	129 ± 51[ab]	112 ± 19[b]	**0.022**	**0.046**
**HbA1c (mmol/mol)**	5.04 ± 0.25[a]	7.18 ± 1.06[b]	6.39 ± 0.59[b]	**<0.001**	**<0.001**
**Insulin (µUI/mL)**	5.25 ± 2.21[a]	-	20.4 ± 15.3[b]	**0.024**	**0.004**
**HOMA-IR**	1.10 ± 0.50[a]	-	5.71 ± 4.37[b]	**0.017**	**0.046**
**TC (mg/dL)**	178 ± 16[a]	158 ± 40[b]	127 ± 36[b]	**0.006**	**0.019**
**HDLc (mg/dL)**	61 ± 10	59 ± 17	46 ± 14	0.053	0.086
**LDLc (mg/dL)**	103 ± 10[a]	78 ± 28[b]	53 ± 29[c]	**<0.001**	**0.001**
**TG (mg/dL)**	66 (41, 73)[a]	90 (77, 104)[a]	122 (77, 203)[b]	**0.003**	**0.007**
**hsCRP (mg/dL)**	0.410 (0.29, 0.73)[a]	2.56 (1.20, 3.77)[b]	1.63 (0.84, 3.00)[b]	**0.011**	0.076
**C3c (mg/dL)**	100 ± 10[a]	115 ± 18[a]	135 ± 9[b]	**<0.001**	**<0.001**
**RBP4 (mg/dl)**	1.76 ± 0.28	1.99 ± 0.69	2.28 ± 0.44	0.069	0.187
**AIP**	-0.342 ± 0.168[a]	-0.194 ± 0.255[a]	0.098 ± 0.327[b]	**0.003**	**0.008**
**Leukocytes (×10^9/L)**	5.44 ± 1.07	6.66 ± 1.43	7.08 ± 1.85	0.054	0.110

	Healthy	T1D	T2D	p-value	p-value adjusted by age
**Neutrophils ($\times 10^9$/L)**	3.06 ± 0.89	3.76 ± 0.94	4.11 ± 1.21	0.088	0.155

Table. S1. Continuation.

Medication	Healthy	T1D	T2D	p-value
**Insulin**	-	100%	50%	**0.033**
**DPP4 inhibitors**	-	22.2%	20%	0.906
**Glidine**	-	11.1%	10%	0.937
**Glitazones**	-	0.00%	20%	0.474
**Metformin**	-	22.2%	80%	0.158
**SGLT2 inhibitors**	-	0.00%	80%	**0.001**
**Statins**	-	55.6%	90%	0.141
**GLP-1 analogues**	-	0.00%	50%	**0.033**
**Ezetimibe**	-	33.3%	10%	0.303
**Antihypertensives**	-	33.3%	42.1%	0.650

Data are presented as mean ± standard deviation for parametric variables or as median (25th–75th percentiles) for non-parametric variables. Non-parametric variables were log-transformed prior to analysis to meet the assumptions of the corresponding statistical tests. Group comparisons were performed using one-way ANOVA followed by the Student–Newman–Keuls post hoc test. p-values represent differences between groups and the adjusted p-value represents differences between groups with adjustment for age as a covariate. Percentages of medication used were compared between groups using the Chi-square test.

Abbreviations: Atherogenic index of plasma (AIP) calculated as log TG/ HDLc; Body mass index (BMI) calculated as weight (kg)/ height (m^2); Complement C3 (C3c); Dipeptidyl Peptidase-4 (DPP4); Glycated Hemoglobin (HbA1c); Glucagon-Like Peptide-1 (GLP-1); High-density lipoprotein (HDLc); Homeostasis model assessment of insulin resistance (HOMA-IR); Interleukin-8 (IL-8); Low-density lipoprotein (LDLc); Sodium-Glucose Cotransporter-2 (SGLT2); Type 1 diabetes (T1D); Triglycerides (TG); Type 2 diabetes (T2D); High sensitivity C reactive protein (hsCRP); Retinol-binding protein 4 (RBP4).

Table S2. Classical leukocyte-endothelium interaction parameters obtained through Tracker v6.1.5.

Type	Patient ID	Rolling velocity (µm/s)	Rolling flux (cells/min)	Adhesion (cells/mm^2)
Healthy	3679	979.40	98	0.25
	3738	1169.34	48	0.12
	3741	1022.77	53	0.18
	3744	970.10	66	0.25
	3812	977.69	56	0.20
	3816	1016.41	47	0.50
	3858	864.40	39	0.10
	3860	855.50	53	0.30
	3861	778.60	65	0.40
	4616	804.32	69	0.10
T1D	3776	446.40	94	0.22
	3737	648.46	270	0.27
	3760	638.00	71	0.33
	3761	422.25	157	1.33
	3774	813.93	109	0.25
	3775	452.88	79	0.16
	3876	625.00	143	1.18
	3878	483.53	127	0.33
	3772	465.30	198	1.25
	4787	463.50	200	1.00
T2D	3748	439.91	178	1.18
	3755	940.80	157	0.33
	3777	728.60	111	0.36
	3809	479.55	190	0.80
	3811	895.10	111	0.70
	3821	860.40	115	0.90
	3835	803.40	121	0.50
	3836	781.00	116	0.70
	3840	772.30	106	0.75
	3821	860.40	107	0.80
Healthy+TNF-α	3679	914.38	126	0.66
	3738	925.97	67	1.07
	3741	930.08	58	0.63
	3744	809.10	74	0.72
	3812	878.62	61	1.10
	3816	956.09	55	1.30
	3858	793.50	46	0.25
	3860	718.90	70	0.75
	3861	610.60	85	0.92
	4616	673.63	122	0.45
T1D+TNF-α	3776	403.55	147	0.45
	3737	520.24	316	2.40
	3760	485.00	200	0.38
	3761	394.24	180	2.31

	3774	679.53	241	0.33
	3775	438.15	107	0.25
	3876	270.00	191	2.27
	3878	422.94	166	0.44
	3772	447.70	210	1.25
	4787	358.60	200	1.29
T2D+TNF-α	3748	314.94	180	2.27
	3755	932.50	149	0.72
	3777	623.70	175	0.81
	3809	417.29	210	1.80
	3811	799.00	175	1.54
	3821	779.60	165	1.60
	3835	778.96	233	1.57
	3836	689.97	192	2.17
	3840	697.31	226	2.35
	3852	497.50	135	2.41

Abbreviations: Identification (ID); Type 1 Diabetes (T1D); Type 2 Diabetes (T2D); Tumor Necrosis Factor Alpha (TNF-α).

Table S3. *p*-values obtained through the HSD Tuckey post hoc test between different groups using rolling velocity as a discriminatory parameter. Groups showing statistically significant differences are highlighted in bold.

Group	Healthy	Healthy+ TNF-α	T1D	T1D+ TNF-α	T2D
**Healthy+TNF-α**	0.397	-	-	-	-
**T1D**	**$1.0 \cdot 10^{-6}$**	**$9.1 \cdot 10^{-4}$**	-	-	-
**T1D+TNF-α**	**$2.3 \cdot 10^{-9}$**	**$2.9 \cdot 10^{-6}$**	**$9.1 \cdot 10^{-4}$**	-	-
**T2D**	0.051	0.909	**0.020**	**$1.1 \cdot 10^{-4}$**	-
**T2D+ TNF-α**	**$4.0 \cdot 10^{-4}$**	0.106	0.549	**0.019**	0.106

Table S4. *p*-values obtained through the HSD Tuckey post hoc test between different groups using rolling flux as a discriminatory parameter. In bold are indicated those two groups with statistically differences.

Group	Healthy	Healthy+ TNF-α	T1D	T1D+ TNF-α	T2D
**Healthy+TNF-α**	0.938	-	-	-	-
**T1D**	**$3.0 \cdot 10^{-4}$**	**0.006**	-	-	-
**T1D+TNF-α**	**$1.2 \cdot 10^{-8}$**	**$3.9 \cdot 10^{-7}$**	0.077	-	-
**T2D**	**0.003**	**0.046**	0.976	**0.011**	-
**T2D+TNF-α**	**$1.3 \cdot 10^{-7}$**	**$4.1 \cdot 10^{-6}$**	0.285	0.987	0.061

Abbreviations: Type 1 Diabetes (T1D); Type 2 Diabetes (T2D); Tumor Necrosis Factor Alpha (TNF-α).

Table S5. *p*-values obtained through the HSD Tuckey post hoc test between different groups using adhesion as a discriminatory parameter. In bold are indicated those two groups with statistically differences.

Group	Healthy	Healthy+ TNF-α	T1D	T1D+ TNF-α	T2D
**Healthy+TNF-α**	0.187	-	-	-	-
**T1D**	0.537	0.985	-	-	-
**T1D+TNF-α**	**0.004**	0.649	0.259	-	-
**T2D**	0.353	0.999	0.999	0.421	-
**T2D+TNF-α**	**$4.9 \cdot 10^{-7}$**	**0.002**	**$2.2 \cdot 10^{-4}$**	0.649	**$6.3 \cdot 10^{-4}$**

Abbreviations: Type 1 Diabetes (T1D); Type 2 Diabetes (T2D); Tumor Necrosis Factor Alpha (TNF-α).

Table S6. Model variables and results obtained from LDA for a 1 *vs* 1, 1 *vs* 1 *vs* 1 and all *vs* all data comparison obtained from classical parameters.

**Model**	**Introduced variables (standarized coeficients: F1; F2: F3)**	λ_{Wilks}	**Classification results**	
Healthy *Vs* Healthy+TNF-α	Rolling velocity (-0.488)<br>Rolling flux (-0.319)<br>Adhesion (0.948)	0.304	Original<br>Healthy: 100%<br>Healthy+TNF-α : 90%	Cross-validation<br>Healthy: 90%<br>Healthy+TNF-α: 90%
Healthy *Vs* T1D	Rolling velocity (0.885)<br>Rolling flux (-0.510)<br>Adhesion (0.115)	0.211	Original<br>Healthy: 100%<br>T1D: 90%	Cross-validation<br>Healthy: 100%<br>T1D: 90%
Healthy+TNF-α *Vs* T1D	Rolling velocity (0.803)<br>Rolling flux (-0.532)<br>Adhesion (0.322)	0.340	Original<br>Healthy+TNF-α: 90%<br>T1D: 80%	Cross-validation<br>Healthy+TNF-α: 80%<br>T1D: 80%
Healthy *Vs* T2D	Rolling velocity (0.392)<br>Rolling flux (0.897)<br>Adhesion (0.615)	0.232	Original<br>Healthy: 100%<br>T2D: 90%	Cross-validation<br>Healthy: 90%<br>T2D: 90%

Healthy+TNF-α<br>*Vs*<br>T2D	Rolling velocity (0.409)<br>Rolling flux(1.157)<br>Adhesion (-0.108)	0.474	Original	Cross-validation
			Healthy+TNF-α: 80%<br>T2D: 100%	Healthy+TNF-α: 80%<br>T2D: 80%
T1D<br>*Vs*<br>T2D	Rolling velocity (1.058)<br>Rolling flux (-0.091)<br>Adhesion (0.550)	0.582	Original	Cross-validation
			T1D: 80%<br>T2D: 80%	T1D: 70%<br>T2D: 80%
Healthy<br>*Vs*<br>T1D<br>*Vs*<br>T2D	Rolling velocity (0.780; 0.673)<br>Rolling flux (-0.485; 0.418)<br>Adhesion (0.033; 0.796)	0.803	Original	Cross-validation
			Healthy: 100%<br>T1D: 80%<br>T2D: 80%	Healthy: 100%<br>T1D: 70%<br>T2D: 80%
Healthy+TNF-α<br>*Vs*<br>T1D+TNF-α<br>*Vs*<br>T2D+TNF-α	Rolling velocity (0.770; 0.555)<br>Rolling flux (-0.889; 0.264)<br>Adhesion (0.384; 0.863)	0.668	Original	Cross-validation
			Healthy+TNF-α: 100%<br>T1D+TNF-α: 90%<br>T2D+TNF-α: 60%	Healthy+TNF-α: 90%<br>T1D+TNF-α: 80%<br>T2D+TNF-α: 60%

Healthy/ Healthy+TNF-α<br>*Vs*<br>T1D/T1D+TNF-α<br>*Vs*<br>T2D/T2D+TNF-α	Rolling velocity (0.820; 0.599)<br>Rolling flux (-0.741; 0.477)<br>Adhesion (0.550; 0.762)	0.781	Original<br>Healthy/Healthy+TNF-α: 95%<br>T1D/T1D+TNF-α: 75.0%<br>T2D/T2D+TNF-α: 55%	Cross-validation<br>Healthy/ Healthy+TNF-α: 85%<br>T1D/T1D+TNF-α: 75%<br>T2D/T2D+TNF-α: 55%
Healthy<br>*Vs*<br>Healthy+TNF-α<br>*Vs*<br>T1D<br>*Vs*<br>T2D<br>*Vs*<br>T1D+TNF-α<br>*Vs*<br>T2D+TNF-α	Rolling velocity (0.686; 0.559; 0.567)<br>Rolling flux (-0.716; 0.048; 0.777)<br>Adhesion (0.076; 1.001; -0.468)	0.927	Original<br>Healthy: 90%<br>Healthy+TNF-α:: 60%<br>T1D: 30%<br>T1D+TNF-α: 50%<br>T2D: 50%<br>T2D+TNF-α: 70%	Cross-validation<br>Healthy: 90%<br>Healthy+TNF-α:: 50%<br>T1D: 30%<br>T1D+TNF-α: 20%<br>T2D: 50%<br>T2D+TNF-α: 60%

Abbreviations: Linear discriminant analysis (LDA); Type 1 Diabetes (T1D); Type 2 Diabetes (T2D); Tumor Necrosis Factor Alpha (TNF-α).

Table S7. Average ± SD values of intracellular ROS production and mitochondrial function markers in neutrophils, together with circulating biomarkers of innate immune activation, endothelial activation, leukocyte recruitment, and cytokine-mediated inflammatory signaling healthy, TID and T2D subjects.

Parameter	Healthy	T1D	T2D	Parameter	Healthy	T1D	T2D
**tROS (DCFH RFU)**	2000 ± 1000	2000 ± 800	2000 ± 600	**IFN-γ (ng/ml)**	44 ± 17	70 ± 40	28 ± 23
**mtMass (MTG RFU)**	13000 ± 13000	12000 ± 4000	12000 ± 7000	**IL-10 (ng/ml)**	30 ± 40	50 ± 70	13 ± 12
**mtROS (MTX RFU)**	580 ± 230	600 ± 130	800 ± 300	**IL-12 (ng/ml)**	5 ± 2	5 ± 3	2.7 ± 1.5
**Superoxide (dHE RFU)**	2200 ± 800	3200 ± 1200	3200 ± 1500	**IL-13 (ng/ml)**	20 ± 20	24 ± 30	8 ± 8
**MMP (TMRM RFU)**	110000 ± 160000	120000 ± 110000	140000 ± 140000	**IL-1β (ng/ml)**	2.5 ± 1.3	3 ± 2	1.5 ± 0.8
**MPO (ng/ml)**	880 ± 990	2300 ± 1600	1300 ± 900	**IL-2**	8 ± 5	7 ± 4	3.4 ± 2.5

				**(ng/ml)**			
**sP-selectin (ng/ml)**	900 ± 400	1400 ± 800	1000 ± 700	**IL-4 (ng/ml)**	98 ± 130	130 ± 210	10 ± 24
**sE-selectin (ng/ml)**	170 ± 50	200 ± 30	200 ± 70	**IL-5 (ng/ml)**	9 ± 9	13 ± 15	3 ± 3
**sICAM-1 (ng/ml)**	330 ± 160	500 ± 200	340 ± 180	**IL-6 (ng/ml)**	12 ± 17	15 ± 29	3 ± 2
**sVCAM-1 (ng/ml)**	180 ± 150	180 ± 50	160 ± 40	**IL-7 (ng/ml)**	15 ± 7	16 ± 9	10 ± 2
**GM-CSF (ng/ml)**	7 ± 4	8 ± 3	5 ± 6	**IL-8 (ng/ml)**	23 ± 25	36 ± 42	19 ± 8

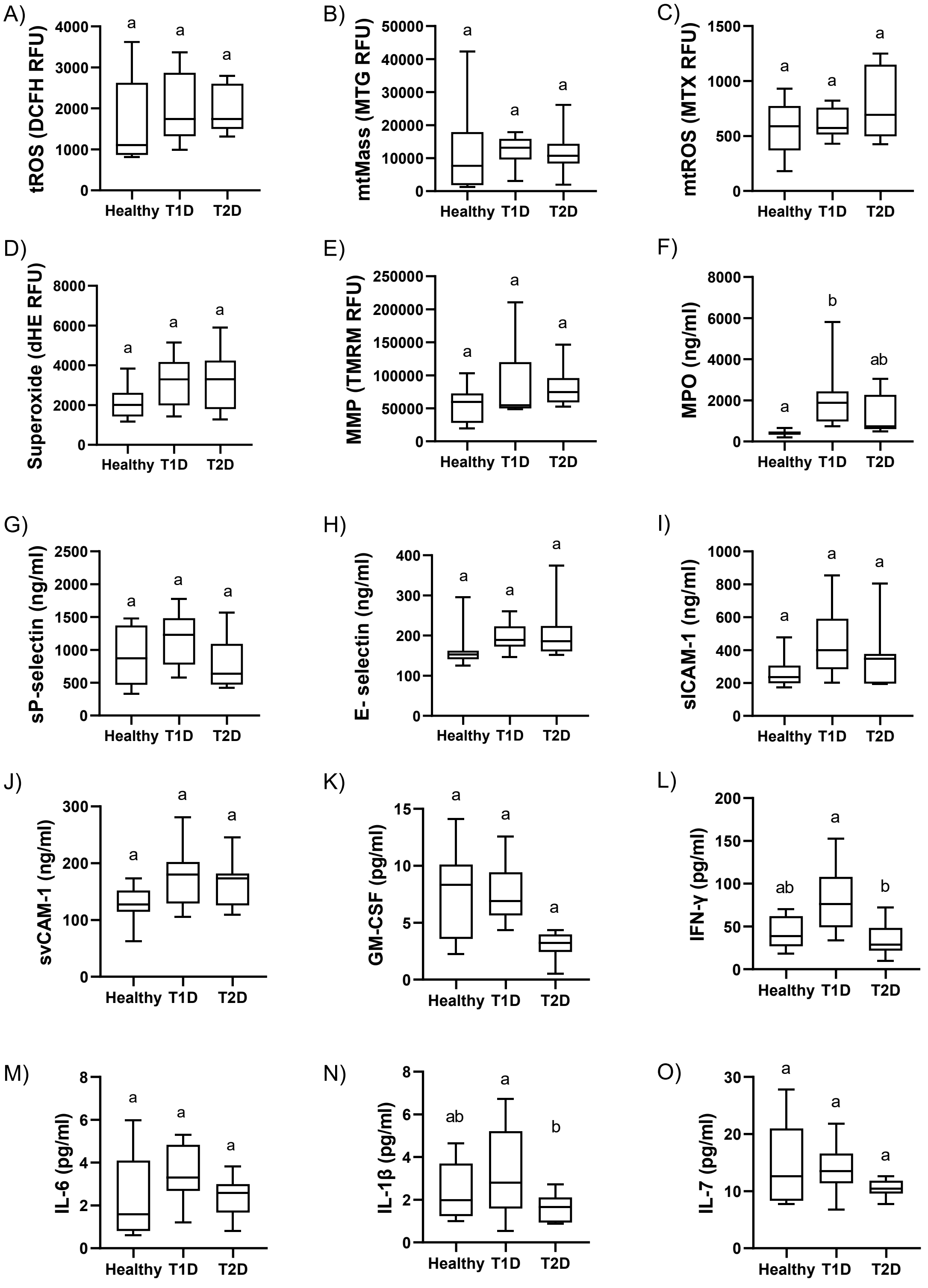

Figure S1. Serum levels of intracellular ROS production and mitochondrial function markers (A-F), adhesion molecules (G-J) and cytokines (K-V) in Healthy, T1D, and T2D subjects. Data are presented as box and whisker plots (min–max), with all individual data points shown. Different superscript letters indicate significant differences among groups (Healthy, T1D, and T2D), as determined by one-way ANOVA and HSD Tukey post hoc. Groups sharing the same superscript letter are not significantly different from each other (p

> 0.05), whereas groups with different letters show statistically significant differences ($p < 0.05$). Abbreviations: Myeloperoxidase (MPO); soluble E-selectin (sE-selentin); soluble intercellular adhesion molecule-1 (sICAM-1); soluble P- Selectin (sP-selectin); soluble vascular cell adhesion molecule-1 (sVCAM-1); granulocyte macrophage colony-stimulating factor (GM-CSF); interferon-gamma (IFN-γ); interleukin (IL); Type 1 diabetes (T1D); Type 2 diabetes (T2D).

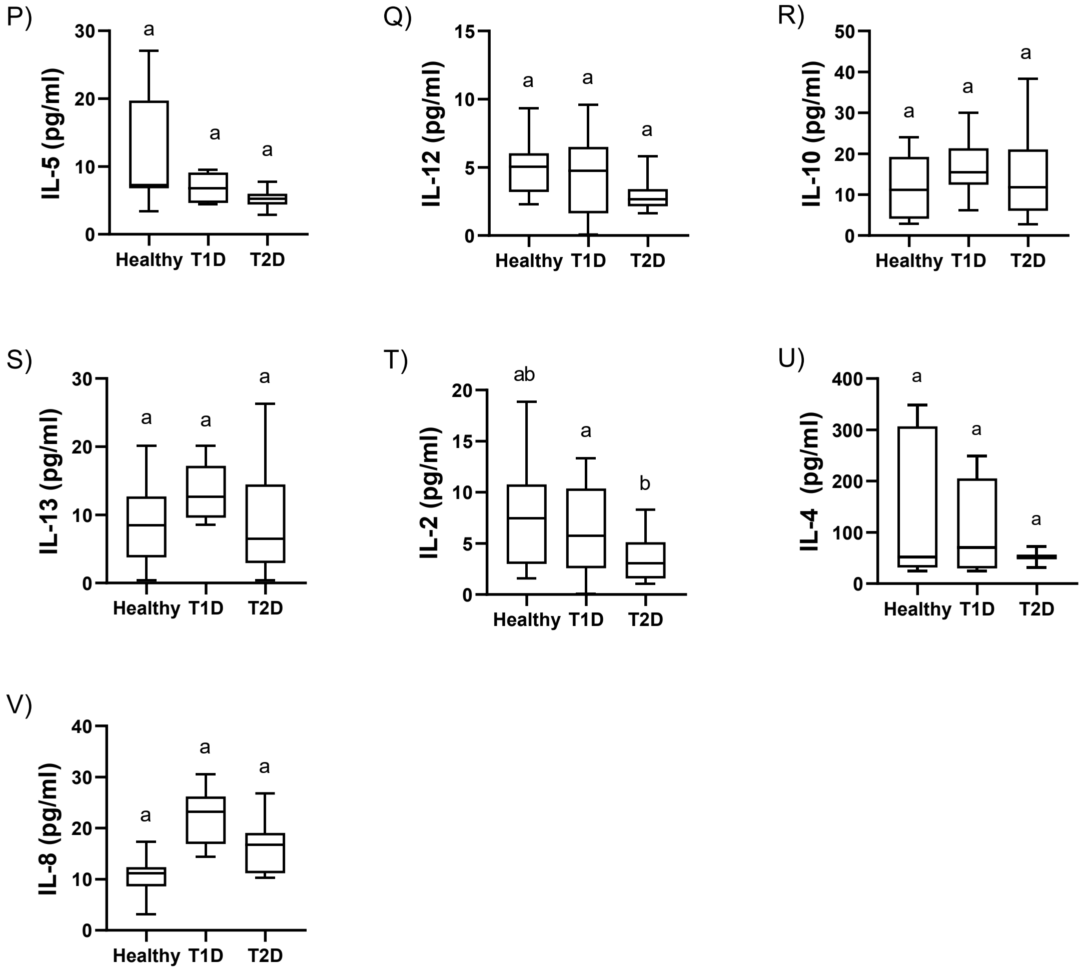

Figure S1 Continuation.

Table S8. *p*-values obtained through the HSD Tuckey post hoc test between different groups using intracellular and circulating biomarkers as discriminatory parameters. In bold are indicated those groups with statistically significant differences.

**Parameter**	**ANOVA**	**Healthy vs T1D**	**Healthy vs T2D**	**T1D vs T2D**	**Parameter**	**ANOVA**	**Healthy vs T1D**	**Healthy vs T2D**	**T1D vs T2D**
**tROS**	0.568	> 0.05	> 0.05	> 0.05	**IFN-γ**	**0.007**	> 0.05	> 0.05	**0.005**
**mtMass**	0.949	> 0.05	> 0.05	> 0.05	**IL-10**	0.323	> 0.05	> 0.05	> 0.05
**mtROS**	0.157	> 0.05	> 0.05	> 0.05	**IL-12**	0.058	> 0.05	> 0.05	> 0.05
**Superoxide**	0.098	> 0.05	> 0.05	> 0.05	**IL-13**	0.243	> 0.05	> 0.05	> 0.05
**MMP**	0.870	> 0.05	> 0.05	> 0.05	**IL-1β**	**0.037**	> 0.05	> 0.05	**0.028**
**MPO**	**0.048**	**0.044**	> 0.05	> 0.05	**IL-2**	**0.044**	> 0.05	> 0.05	**0.040**
**sP-selectin**	0.257	> 0.05	> 0.05	> 0.05	**IL-4**	0.167	> 0.05	> 0.05	> 0.05
**sE-selectin**	0.234	> 0.05	> 0.05	> 0.05	**IL-5**	0.132	> 0.05	> 0.05	> 0.05
**sICAM-1**	0.107	> 0.05	> 0.05	> 0.05	**IL-6**	0.365	> 0.05	> 0.05	> 0.05
**sVCAM-1**	0.855	> 0.05	> 0.05	> 0.05	**IL-7**	0.184	> 0.05	> 0.05	> 0.05
**GM-CSF**	0.305	> 0.05	> 0.05	> 0.05	**IL-8**	0.381	> 0.05	> 0.05	> 0.05

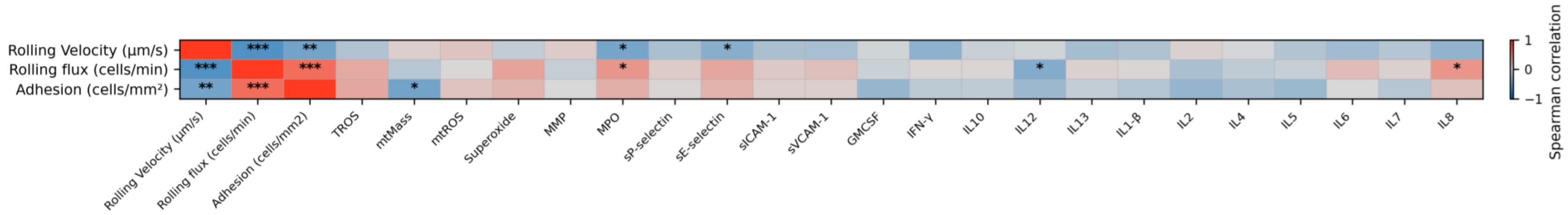

Fig. S2. Heat map of Spearman coefficients for the comparison between circulating biomarkers and classical hydrodynamic variables. *, ** and ** indicates *p*-values lower than 0.05, 0.01 and 0.001, respectively.

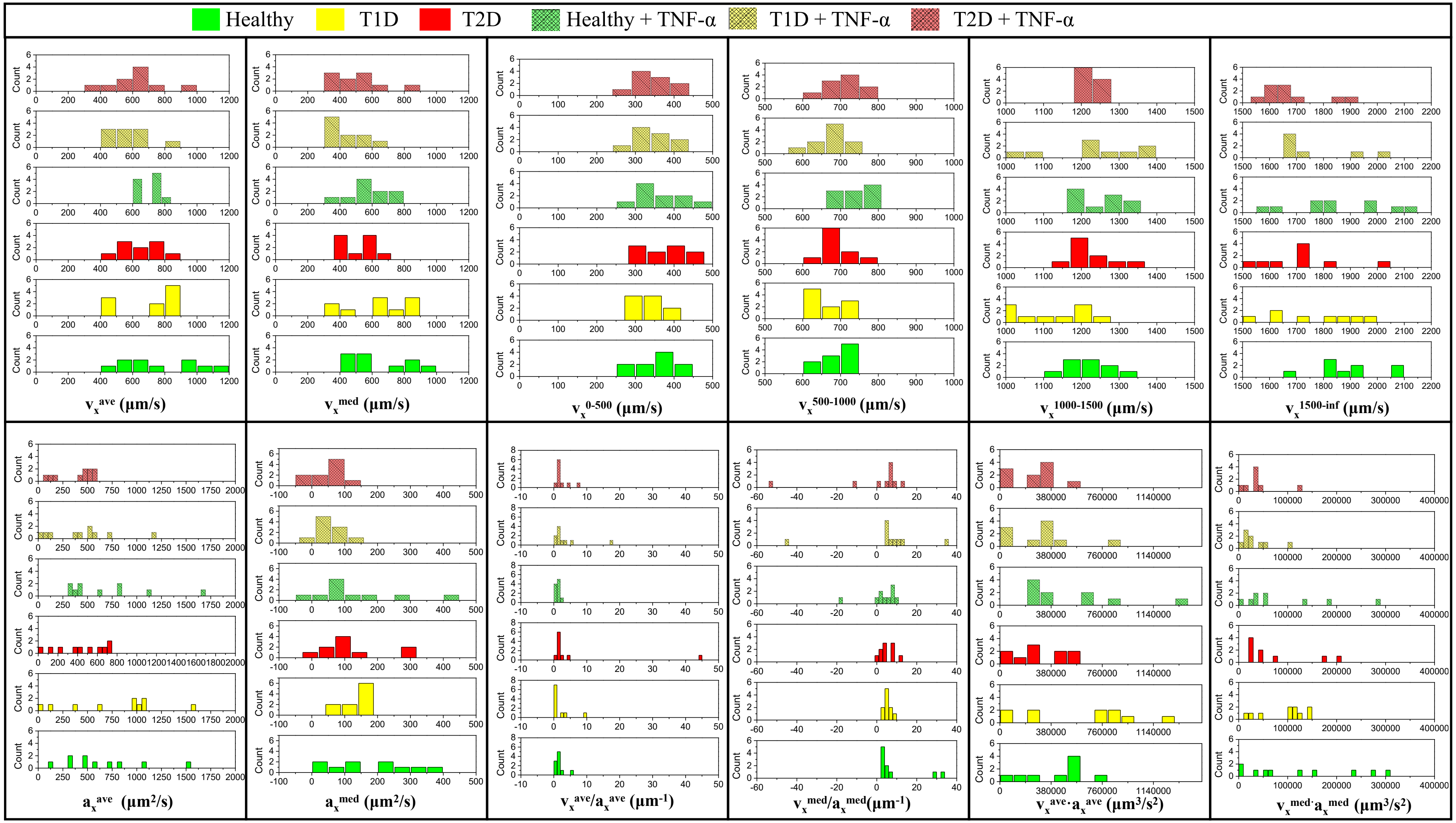

Fig. S3. Histograms of the newly proposed hydrodynamic parameters. Counts means patients for all figures except for X and A where means number of leucocytes.

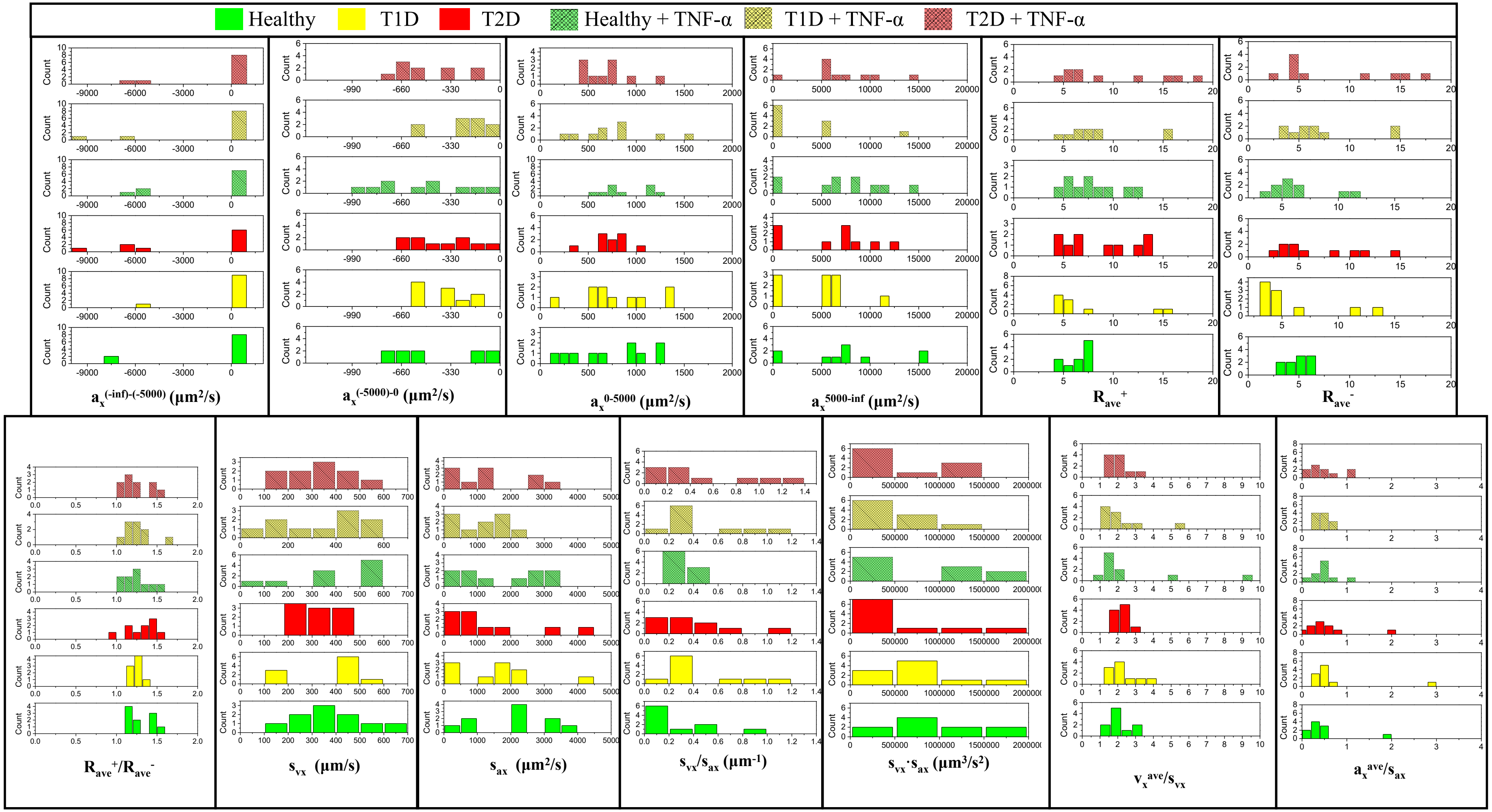

Fig. S3. Continuation

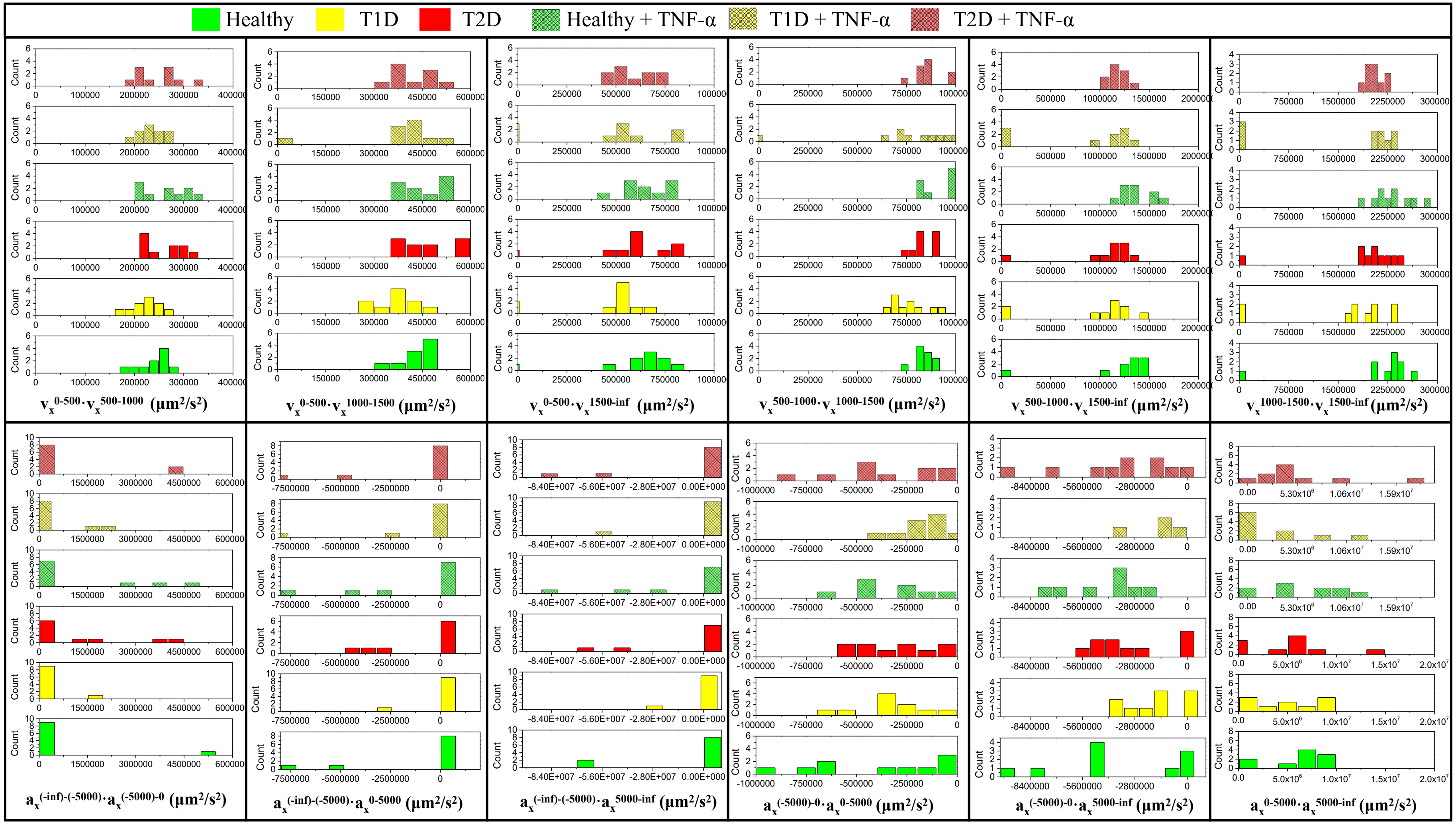

Fig. S3. Continuation

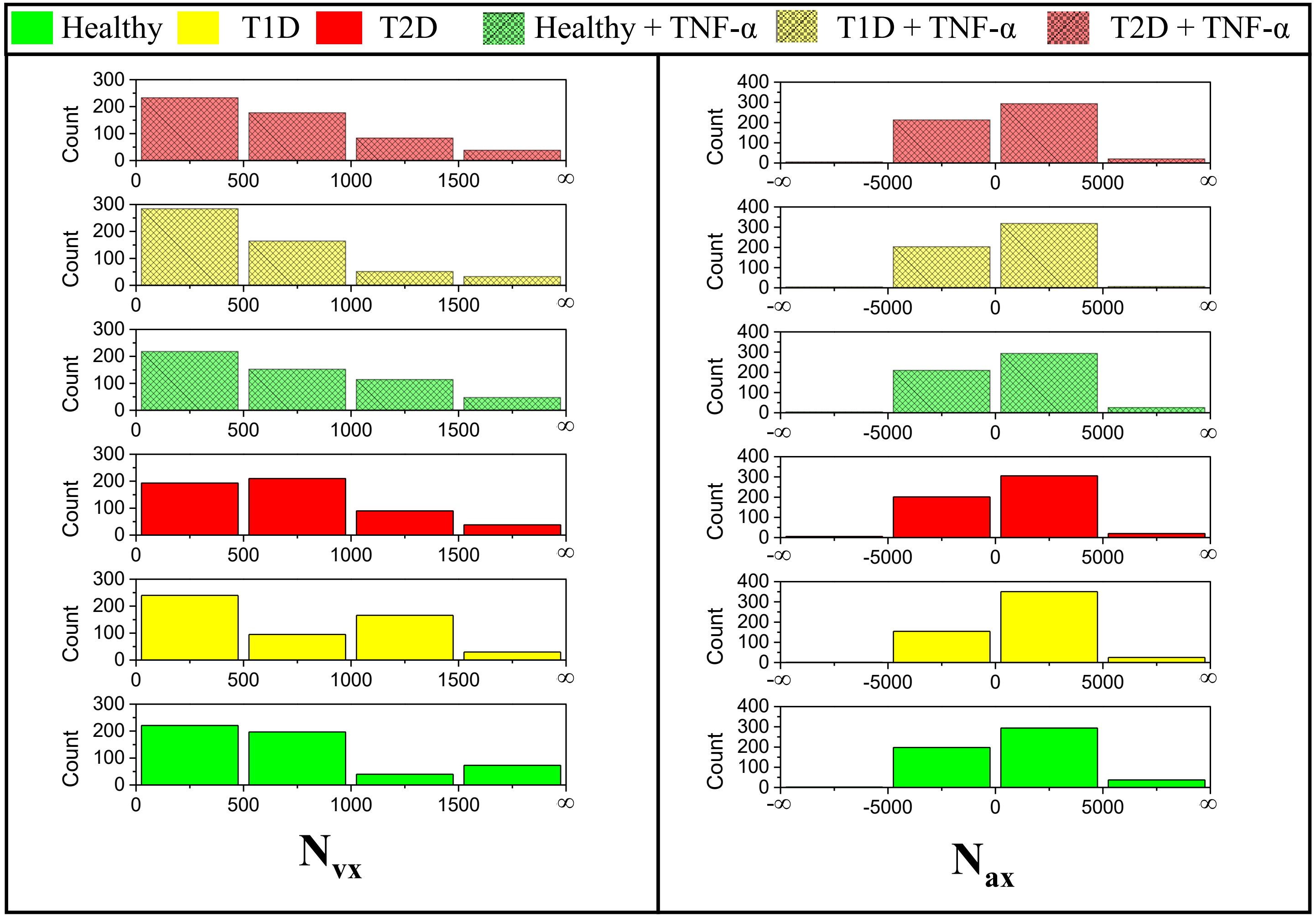

Fig. S3. Continuation

Table S9. Newly introduced hydrodynamic parameters of the study population.

See Excel in the supplementary information named "Tables S9, S10, S11, S12 and S13 - Hermenejildo et al.xlsx".

Table S10. *p*-values obtained through ANOVA and the HSD Tuckey post hoc test between different groups using the hydrodynamic parameters as discriminatory parameters. In bold are indicated those groups with statistically significant differences.

See Excel in the supplementary information named "Tables S9, S10, S11, S12 and S13 - Hermenejildo et al.xlsx".

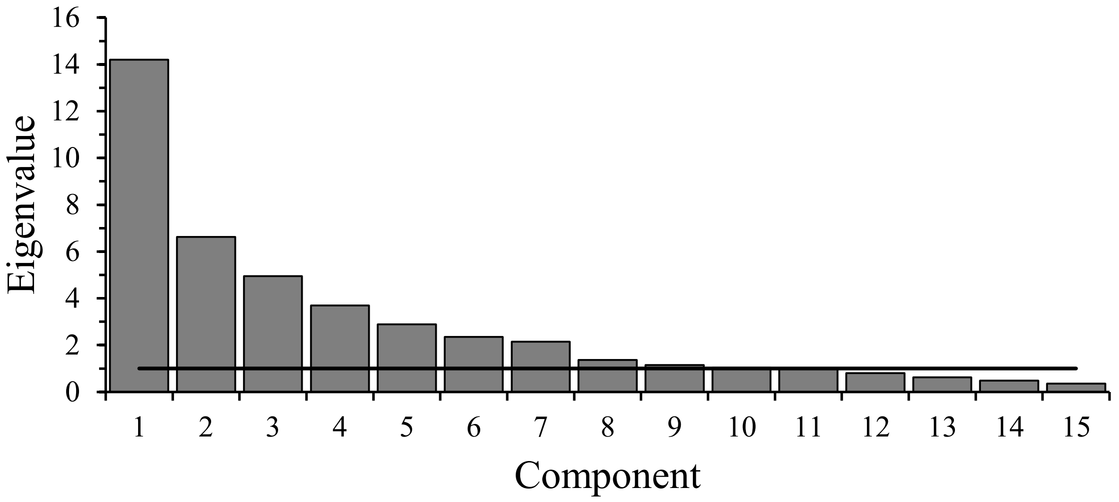

Fig. S4. Scree plot showing the eigenvalues associated with each principal component derived from the PCA of the hydrodynamic variables. The horizontal line indicates the Kaiser criterion (eigenvalue = 1).

Table S11. Varimax-rotated component matrix obtained from the PCA of hydrodynamic variables. Loadings represent correlations between variables and principal components.

See Excel in the supplementary information named "Tables S9, S10, S11, S12 and S13 - Hermenejildo et al.xlsx".

Table S12. Pearson correlation coefficients (r) between circulating biochemical biomarkers and the complete set of hydrodynamic variables derived from leukocyte–endothelium interaction analysis. Correlations were calculated across all subjects (n = 30). Positive and negative values indicate direct and inverse linear associations, respectively.

See Excel in the supplementary information named "Tables S9, S10, S11, S12 and S13 - Hermenejildo et al.xlsx".

Table S13. Spearman rank correlation coefficients (ρ) between circulating biochemical biomarkers and the complete set of hydrodynamic variables derived from leukocyte–endothelium interaction analysis. Correlations were calculated across all subjects (n = 30). Positive and negative values indicate direct and inverse monotonic associations, respectively.

See Excel in the supplementary information named "Tables S9, S10, S11, S12 and S13 - Hermenejildo et al.xlsx".
